# Anti-Inflammatory and Antimicrobial Activities of Compounds Isolated from *Distichochlamys benenica*

**DOI:** 10.1155/2021/6624347

**Published:** 2021-04-06

**Authors:** Ty Viet Pham, Hanh Nhu Thi Hoang, Hoai Thi Nguyen, Hien Minh Nguyen, Cong Thang Huynh, Thien Y Vu, Anh Thu Do, Nguyen Hoai Nguyen, Bich Hang Do

**Affiliations:** ^1^Faculty of Chemistry, University of Education, Hue University, 34 Le Loi, Hue City, Vietnam; ^2^Faculty of Engineering and Food Technology, Hue University of Agriculture and Forestry, Hue University, 102 Phung Hung, Hue City, Vietnam; ^3^Faculty of Pharmacy, University of Medicine and Pharmacy, Hue University, 06 Ngo Quyen, Hue City, Vietnam; ^4^Faculty of Pharmacy, Ton Duc Thang University, Ho Chi Minh City, Vietnam; ^5^English Faculty, Foreign Trade University-Ho Chi Minh City Campus, Vietnam; ^6^Faculty of Biotechnology, Ho Chi Minh City Open University, Ho Chi Minh City, Vietnam

## Abstract

*Distichochlamys benenica* is a native black ginger that grows in Vietnam. In point of fact, there is limitation of available information in the literature making mention of the chemical constituents and bioactive properties of this plant. This study is aimed at isolating *trans-o*-coumaric acid (**1**), *trans*-cinnamic acid (**2**), and borneol (**3**) from the rhizomes of *D. benenica* Q.B.Nguyen & Škorničk and evaluate the anti-inflammatory and antimicrobial activities of **1-3** using the carrageenan paw edema model and the dilution broth method, respectively. This revealed that **1** was as effective as diclofenac in reducing the intensity of the edema development. The *in silico* research showed that the activity of **1** might be derived from inhibiting COX-2 by generating h-bonds at the positions of Arg 120, Tyr 355, and Arg 513 residues. The antimicrobial activities against Gram-positive strains (*Staphylococcus aureus* and *Bacillus subtilis*) were comparable, with the minimum inhibitory concentrations ranging from 1.52 to 3.37 mM. This is the first study of the bioactivity of compounds isolated from *D. benenica* Q.B.Nguyen & Škorničk. Our results suggest that **1** may be a nature-derived compound which demonstrates the anti-inflammatory properties and inhibit the proliferation of several Gram-positive bacteria.

## 1. Introduction

Inflammation is an immune response to pathogen invasion that is mediated by the release of mediators including cytokines, histamine, prostaglandin, and leukotrienes, as well as enhanced vascular permeability, leading to the recruitment of leukocytes to the site of inflammation, resulting in the clearing of pathogens [1, 2]. Nevertheless, if inflammation is not regulated properly, it may cause different diseases including autoimmune, neurodegenerative, and cardiovascular diseases, cancer, diabetes mellitus, and diseases [2–4]. Hence, compounds which enable amelioration of the inflammation can enhance the efficiency in treating those diseases. Many plant extracts and their isolated compounds are effective at reducing inflammatory mediators, recruiting leukocytes, moderating swelling, and alleviating inflammatory symptoms [5–7]. Additionally, plants are also valuable sources of antimicrobials [8, 9]. With the development of antibiotic resistance, alternative antimicrobial compounds are urgently needed.

The genus *Distichochlamys* (Zingiberaceae) was discovered in Bach Ma National Park, Thua Thien Hue Province, Vietnam, in 1995 by M. F. Newman [10]. Traditionally, this genus is used by ethnic Pako as food and as medicine for abdominal pain, blood coagulation, wound healing, and pus removal. Four congenerics have been identified: D. *citrea* M.F. Newman, D. *rubrostriata* W.J. Kress and Rehse, D. *benenica* Q.B. Nguyen & Škorničk, and D. *orlowii* K. Larsen and M.F. Newman. The extracts of Zingiberaceae possessed antioxidant, antimicrobial, and anti-inflammatory properties [11–14]. Although the chemical compositions of the *Distichochlamys* genus have been investigated [15–17], few studies have examined the bioactivity. One study mentioned that the essential oil from the rhizomes of D. *benenica* possesses acetylcholinesterase activity [18].

Cinnamic acid, an aromatic acid isolated from natural sources, holds various bioactivities including antidiabetes, anti-inflammatory, antityrosinase, antimicrobial, antioxidant, and anticancer properties [19–21]. Coumaric acid is a hydroxy derivative of cinnamic acid with three isomers: *o*-, *m*-, and *p-*coumaric acids. The most widely studied is *p*-coumaric, which has anti-inflammatory, antioxidant, antibacterial, anticancer, and diabetes mitigation effects [22–25]. *Trans-o-*coumaric acid was demonstrated to have antimicrobial and antifungal abilities [26]. Borneol is a naturally occurring bicyclic monoterpenoid that possesses antimicrobial and anti-inflammatory properties and increases blood-brain permeability, enhancing the enhancement of drug delivery to the central nervous system [27–30].

In this study, we isolated *trans-o*-coumaric acid (**1**) [26], *trans*-cinnamic acid (**2**) [31], and borneol (**3**) [32] from the rhizomes of *D. benenica* Q.B.Nguyen & Škorničk, elucidated their structures, and investigated their bioactive properties. For the first time, the bioactivities of compounds isolated from *D. benenica* have been investigated. The anti-inflammatory activity was examined *in vivo* using a carrageenan-induced edema assay, and the antimicrobial activity was evaluated using the broth dilution test.

## 2. Materials and Methods

### 2.1. Materials

Diclofenac, carrageenan, and solvents such as *n*-hexane, chloroform, acetone, ethyl acetate, and methanol were obtained from Sigma-Aldrich (St Louis, MO, USA). Mueller-Hinton Broth was purchased from Thermo Fisher Scientific (Massachusetts, USA). Silica gel (60 N, spherical, neutral, 40-50 *μ*m) was obtained from Kanto Chemical Co., Inc. (Tokyo, Japan). Plethysmometer was purchased from Panlab, Havard Apparatus. Infrared spectra were recorded on an IR Prestige-21 spectrometer (Shimadzu, Kyoto, Japan). NMR spectra were recorded using a Bruker Avance 500 spectrometer (Bruker, MA, USA), with TMS as an internal reference. HRESIMS data were measured on an Agilent 6530 Accurate-Mass spectrometer (Agilent, CA, USA). Analytical TLC was performed on precoated silica gel 60F_254_ and RP-18 F_254_ plates (0.25 or 0.50 mm thickness, Merck KGaA, Darmstadt, Germany). Spots were detected under UV radiation (254 nm and 365 nm) and by spraying the plates with 10% sulfuric acid followed by heating with a heat gun.

*D. benenica* Q.B.Nguyen & Škorničk was collected in January 2020 at Quang Nam province and identified by Hue University of Agriculture and Forestry. A voucher specimen (DB1) was deposited in the Faculty of Chemistry at the Hue University of Education.

### 2.2. Methods

#### 2.2.1. The Preparation of Compounds

The rhizomes of *D. benenica* were dried and ground before applying to the extraction process. The rhizomes (1.2 kg) of *D. benenica* were powdered and extracted with *n*-hexane (2.5 L × 5 cycles), chloroform (2.0 L × 5 cycles), and methanol (2.0 L × 4 cycles) at room temperature. The supernatants were evaporated at under 40°C *in vacuo* to obtain three extracts including crude *n*-hexane (DBH, 80.0 g), crude chloroform (DBC, 85.0 g), and crude methanol (DBM, 105.0 g), respectively. The DBH extract (80.0 g) was applied to column chromatography on silica gel and subsequently eluted with a gradient system of *n*-hexane-acetone (100 : 0, 40 : 1, 20 : 1, and 10 : 1, *v*/*v*, 2.0 L each) to obtain 4 fractions (H1-H4). Fraction H3 (5.0 g) was chromatographed on a silica gel column eluted with *n*-hexane-acetone (95 : 5, *v*/*v*) to yield borneol (**3**) (21.8 mg). Similarly, fraction H4 (7.5 g) was chromatographed on a silica gel column eluted with *n*-hexane-EtOAc (90 : 10, *v*/*v*) to give *trans*-cinnamic acid (**2**) (16.9 mg) and *trans-o*-coumaric acid (**1**) (13.7 mg), respectively. Compounds **1-3** were dissolved in DMSO to the concentration of 100 mg/mL then diluted into 0.9% NaCl to the tested concentrations. The samples were filtered through 0.4 *μ*m filters before use.

#### 2.2.2. Experimental Mice

Swiss albino mice of 6-8 weeks of age (18 ± 2 g) were obtained from Pasteur Institute, Ho Chi Minh City, Vietnam. They were housed under standard husbandry conditions with a 12 h light-dark cycle for at least 1 week to acclimate with the laboratory environment. They were supplied ad libitum with standard chow and distilled water. The healthy mice were used in the experiments. The experimental procedure was strictly complied with the Declaration of Helsinki (1964).

#### 2.2.3. The Anti-Inflammation Assay

The anti-inflammatory ability of **1-3** was evaluated using the carrageenan-induced paw edema method following the previously described protocol [33]. The mice were divided into 5 groups of 6 mice each. Mice were intraperitoneally pretreated with **1-3** at a dose of 20 mg/kg. Mice treated with saline (0.9% w/v NaCl, p.o) and diclofenac (10 mg/kg, p.o) were used as negative and positive controls, respectively. One hour later, mice were subplantar injected with 40 *μ*L 1% carrageenan (suspended in saline) into the right hind paw to stimulate the edema. The paw volume was measured using a plethysmometer before carrageenan treatment (*C*_0_) and after carrageenan injection at selected time intervals 1, 2, 3, and 4 h (*C*_*t*_).

The anti-inflammatory activity was calculated using the following equation:

(1) $$\begin{array}{c} \%\text{Inhibition}=100\times \frac{\left({\left(C_{t}-C_{0}\right)}_{\text{control}}-{\left(C_{t}-C_{0}\right)}_{\text{treated}}\right)}{{\left(C_{t}-C_{0}\right)}_{\text{control}}}, \end{array}$$

where (*C*_*t*_ − *C*_0_)_control_ is the difference of the paw volume in the negative control group, and (*C*_*t*_ − *C*_0_)_treated_ is the difference of the paw volume in the tested or positive control groups.

The highest anti-inflammatory compound was confirmed further at different concentrations. The protocol is similar to the aforementioned one. Briefly, the mice were peritoneally injected with the compound at dosages of 5, 10, and 20 mg/kg 1 h before injection of carrageenan into the right hind paw. Mice treated with saline (0.9% _w/v_ NaCl, p.o) and diclofenac (10 mg/kg, p.o) were used as negative and positive controls, respectively. The volume of the paw was assessed before (*C*_0_) and after carrageenan injection (*C*_*t*_) hourly until 4 h using a plethysmometer. The anti-inflammatory activity was determined following the aforementioned equation.

After 4 h of carrageenan treatment, the inflamed paws were pictured to compare the change in appearance of treated groups.

#### 2.2.4. The Antimicrobial Assay

The antimicrobial activity of **1-3** was determined using the broth microdilution method [34]. The test was performed using sterile polystyrene 96-well plates. 100 *μ*L of autoclaved Muller-Hinton Broth supplemented with different concentrations of **1-3** (1, 0.5, 0.25, 0.125, 0.0625, and 0.03125 mg/mL) was added into each well. Subsequently, 5 *μ*L of the bacteria including *Staphylococcus aureus*, *Bacillus subtilis*, *Pseudomonas aeruginosa*, and *Escherichia coli* at the concentration of approximately 10^8^ CFU/mL was added. The plate was shaken and incubated at 37°C for 24 h. 0.1% DMSO was used as a negative control, and ciprofloxacin was used as a positive control. Minimum inhibitory concentration (MIC) was defined as the lowest concentration of the extract at which the microorganisms showed no visible growth.

#### 2.2.5. Ligand Docking

The two targeted proteins used in ligand docking are cyclooxygenase 1 (COX-1) and cyclooxygenase 2 (COX-2), which are the two main proteins responsible for the inflammatory process. The published crystal structures of COX-1 (PDB 2OYU) and COX-2 (PDB 4COX) were imported and prepared by Maestro software (Schrödinger Release 2020-3). The general procedure of the Protein Preparation Wizard [35] was used to assign bond orders, remove waters, generate het states using Epik, optimize the h-bond, and minimize the protein with the OPLS3e force field. The grid box for the docking job was defined by centroid of native ligands in two proteins expanded to 20 Å in a three-dimensional space.

#### 2.2.6. Molecular Dynamics Simulations

Three molecules **1**, **2**, **3** and the reference drug diclofenac were created and prepared by Ligprep [36] to obtain possible ionization states at physiological pH = 7 ± 2. These compounds and the native ligand of crystallized protein were then docked to the grid box of two proteins by using two successive methods Glide [37] SP (standard precision) and XP (extra precision). The refined XP top scores were used to feed the MM-GBSA [38] calculations (molecular mechanics/generalized Born and surface area method) with the VSGB solvation model and same force field. The relative free binding energy values obtained from these calculations are used to compare the inhibition capacity of **1-3** with reference standards. From there, the system set-up of molecular dynamics simulations was built for docked ligands with COX-1 and COX-2 by using Desmond [39]. The solvent model was predefined with SPC, and the force field was kept unchanged OPLS3e. The total simulation time lasted 50 ns for each system, and 50 ps was set to trajectory recording intervals. The system energy was 1.2, and the ensemble class used was NPT at 300.0 K temperature and at 1.01325 bar pressure. The relax model system was a default option before simulations.

#### 2.2.7. Statistical Analysis

All data are presented as the mean ± standard error (SE). Statistical analyses were performed using the SPSS statistical software program (SPSS, version 18.0, Chicago, IL). A Student's *t*-test was used to determine the statistical significance of group means. All tests were two sided, and *p* values less than 0.05 were considered statistically significant.

## 3. Results

### 3.1. The Determination of Isolated Compounds

**Figure 1:**
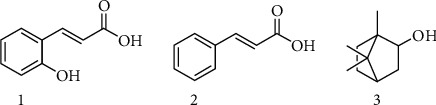
Structures of **1-3** isolated from the rhizomes of *D*. *benenica.*

Compounds **1-3** were isolated from *n-*hexane extract of *D. benenica* Q. B. Nguyen & Škorničk rhizome. Compounds 1 and 2 are white crystalline powder with the molecular formulas of C_9_H_8_O_3_ and C_9_H_8_O_2_, respectively. Compound 3 is white-colored lump-solid with the molecular formula of C_10_H_18_O. The data values of IR, HRESIMS, ^1^H-NMR, and ^13^C-NMR of **1-3** and chemical structures of **1-3** are described in Figure 1 and in Supplementary S1-S22.

*trans-o-*coumaric acid (**1**): IR (KBr) *ν*_max_ (cm^−1^): 3456, 3063, 3028, 2982, 1682, 1628, 1420, 1312, 1285, 1223, 980, 941, 914, 768, 706; HRESIMS *m*/*z* 163.0376 [M-H]^−^; ^1^H-NMR (500 MHz, MeOD): *δ* 7.99 (1H, d, *J* = 16.0 Hz, H-3), 7.49 (1H, d, *J* = 1.0, 8.0 Hz, H-6′), 7.22 (1H, td, *J* =1.0, 8.0 Hz, H-4′), 6.86 (1H, d, *J* = 8.0 Hz, H-3′), 6.85 (1H, t, *J* = 8.0 Hz, H-5′), 6.57 (1H, dd, *J* =16.0 Hz, H-2); ^13^C-NMR (125 MHz, MeOD): *δ* 117.0 (CH, C-2), 118.6 (CH, C-3′), 120.7 (CH, C-5′), 122.6 (C, C-1′), 130.0 (CH, C-6′), 132.5 (CH, C-4′), 142.5 (CH, C-3), 158.2 (C, C-2′), 171.3 (C, C-1).

*trans-*cinnamic acid (**2**): IR (KBr) *ν*_max_ (cm^−1^): 3383, 2951, 2878, 1744, 1632, 1454, 1381, 1304, 1111, 1061, 1022, 941, 829, 663; HRESIMS *m*/*z* 147.0420 [M-H]^−^; ^1^H-NMR (500 MHz, MeOD): *δ* 7.79 (1H, d, *J* = 16.0 Hz, H-3), 7.60 (2H, m, H-2′, 6′), 7.41 (3H, m, H-3′, 4′, 5′), 6.49 (1H, d, *J* = 16.0 Hz, H-2); ^13^C-NMR (125 MHz, MeOD): *δ* 119.4 (CH, C-2), 129.2 (CH, C-2′ and C-6′), 130.0 (CH, C-3′ and C-5′), 131.4 (CH, C-4′), 135.8 (C, C-1′), 146.4 (CH, C-3), 170.4 (C, C-1).

Borneol (**3**): IR (KBr) *ν*_max_ (cm^−1^): 3441, 3364, 3075, 2974, 2843, 1667, 1616, 1458, 1427, 1335, 1219, 1092, 991, 910, 868, 752, 698, 590; HRESIMS *m*/*z* 155.1438 [M + H]^+^; ^1^H-NMR (500 MHz, CDCl_3_): *δ* 4.01 (1H, m, H-2), 2.27 (1H, m, H-4), 1.88 (1H, m, H-4), 1.74 (1H, m, H-3a), 1.73 (1H, m, H-6a), 1.52 (1H, m, H-5a), 1.27 (1H, m, H-6b), 1.25 (1H, m, H-5b), 0.87 (3H, s, H-9), 0.86 (3H, s, H-8) 0.85 (3H, s, H-10); ^13^C-NMR (125 MHz, CDCl_3_): *δ* 13.3 (CH_3_, C-10), 18.7 (CH_3_, C-8), 20.2 (CH_3_, C-9), 25.9 (CH_2_, C-6), 28.3 (CH_2_, C-5), 39.0 (CH_2_, C-3), 45.1 (CH, C-4), 48.0 (C, C-7), 49.5 (C, C-1), 77.3 (CH, C-2).

### 3.2. Anti-Inflammatory Assay

**Figure 2:**
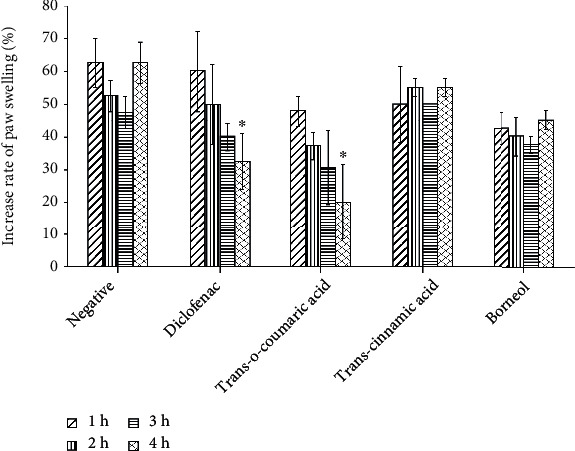
The effect of **1-3** on carrageenan-induced rat paw edema. The mice were peritoneally injected with different compounds, positive or negative control 1 h before induction of inflammation at the right hind paw using 1% carrageenan. The swelling of the paw was measured using a plethysmometer before exposure to the compounds and at 1 h, 2 h, 3 h, and 4 h after treatment of carrageenan. Data are presented as means ± SD (*n* = 6). ^∗^*p* < 0.05, ^∗∗^*p* < 0.01, and ^∗∗∗^*p* < 0.0001 as compared with the negative control group.

To determine the anti-inflammatory activity, an *in vivo* experiment examined acute carrageenan-induced inflammation in mice. Particularly, mice were injected intraperitoneally with **1-3** or positive or negative controls 1 h before inducing inflammation at the right hind paw with 1% carrageenan. The intensity of edema development was measured via plethysmometry. Of the three, **1** significantly reduced edema at a dose of 20 mg/kg, similar to that of the positive control (Figure 2). Four hours after the carrageenan treatment, the paw volume of group 1 mice was reduced by 68% (*p* < 0.05) compared to 48% (*p* < 0.05) for the positive control. The swelling in the negative control group was almost unchanged 4 h after the carrageenan treatment. Similar results were attained in groups **2** and **3** with slight edema suppression, approximately 12% and 28%, respectively (*p* > 0.05). Therefore, **1** was chosen for further study.

**Figure 3:**
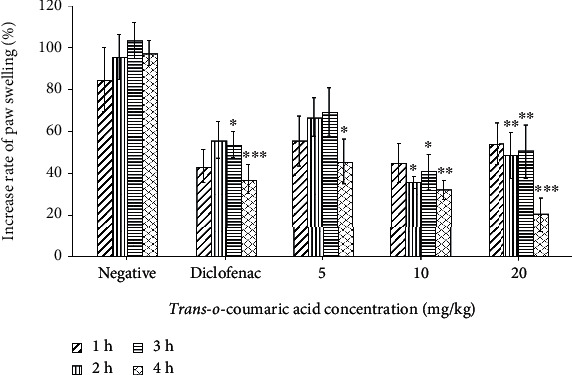
The effect of different concentrations of **1** on carrageenan-induced rat paw edema. The mice were peritoneally injected with **1** at different concentrations (20 mg/kg, 10 mg/kg, and 5 mg/kg), positive or negative control 1 h before induction of inflammation at the right hind paw using 1% carrageenan. The paw edema was measured using a plethysmometer before exposure to the compounds and at 1 h, 2 h, 3 h, and 4 h after treatment of carrageenan. Data are presented as means ± SD (*n* = 6). ^∗^*p* < 0.05, ^∗∗^*p* < 0.01, and ^∗∗∗^*p* < 0.0001 as compared with the negative control group.

The anti-inflammatory bioactivity of **1** at different concentrations was confirmed using a similar method. The mice were injected intraperitoneally with 5, 10, or 20 mg/kg of **1** 1 h before carrageenan was injected to induce inflammation. As displayed in Figure 3, **1** significantly ameliorated the inflammation in a dose-dependent manner. At 5 mg/kg, **1** significantly reduced the edema 4 h after carrageenan treatment (*p* < 0.05). At 10 mg/kg and 20/kg, **1** effectively moderated the paw edema intensity to approximately 67% and 79%, respectively, (*p* < 0.0001), 4 h after carrageenan treatment compared to 62% for the positive control (*p* < 0.0001). There was no significant difference in the paw volume reduction between dosages of 10 mg/kg and 20 mg/kg.

**Figure 4:**
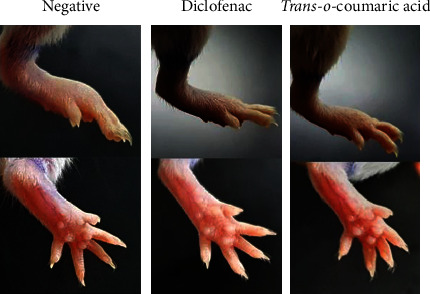
The images of the carrageenan-treated paw after injection of diclofenac or trans-o-coumaric acid. The mice were peritoneally injected with saline (negative), diclofenac (positive), or trans-o-coumaric acid, followed by inflammation induction at the right hind paw using 1% carrageenan. At 4 h after treatment with carrageenan, the treated paws were pictured to compare the size.

The photographs of inflamed mouse paws in groups **1**, the negative control, and the positive control are shown in Figure 4. The reduction in the paw volumes of the mice in group **1** and the positive controls was obvious.

### 3.3. Antimicrobial Assay

To evaluate the antimicrobial ability of **1-3**, different concentrations of **1-3** were added to Gram-positive (*S. aureus*, *B subtilis*) and Gram-negative *(P. aeruginosa, E. coli)* bacterial cultures. After incubation, the MIC was determined and the result is shown (Table 1). Both **1** and **2** comparably inhibited the growth of Gram-positive strains without affecting Gram-negative strains. No obvious bacterial inhibition was observed for **3** against either Gram-positive or Gram-negative strains.

### 3.4. Docking Studies

The RMSD of the native ligands when redocked to COX-1 and COX-2 were 0.56 and 0.45 Å, respectively. These values showed a good binding mode which was reliable for relative free binding energy MM-GBSA postcalculations.

**Figure 5:**
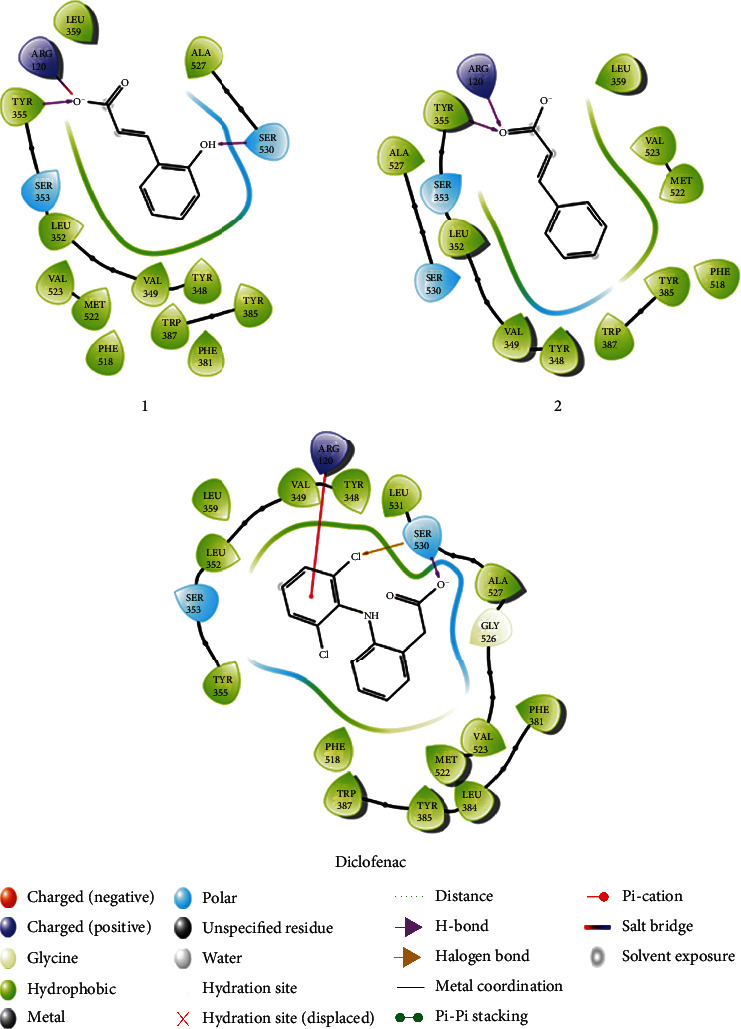
Binding poses of three molecules **1**, **2**, and the diclofenac with COX-2 proteins. The additional information in 2D diagrams was present.

In COX-1 protein, the reference diclofenac had the lowest free energy at -29.3 kcal/mol, and **1** and **2** had higher values at -8.2, and -8.8 kcal/mol, respectively, while **3** was screened out of both COX-1 and COX-2 proteins due to large steric effect structure (Table 2, Supporting S23). In COX-2 protein, diclofenac maintained its best inhibitor activity at –33.9 kcal/mol and molecule **1** showed competitiveness with diclofenac at –29.7 kcal/mol. These estimates were well consistent with *in vivo* results where **1** ameliorated significantly the inflammation similar to diclofenac. Molecule **2** exhibited a poorer inhibitory capacity at –25.2 kcal/mol. In XP docking results, ligand **1** was held tightly by three h-bond donors from the Arg 120, Tyr 355, and Ser 530 residues whereas ligand **2** possessed only two h-bonds donors with Arg 120 and Tyr 355 (Table 2, Figure 5). Taken together, **1** showed a competitive inhibition with diclofenac in COX-2 and has the relative free binding energy similar to diclofenac.

### 3.5. Molecular Dynamics Simulations Predict the Inhibition Mechanism by Molecule 1

**Figure 6:**
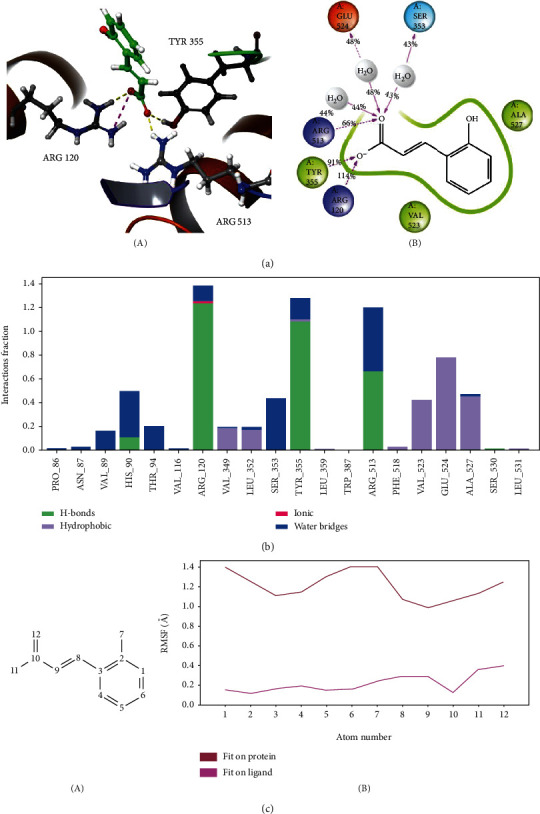
Molecular dynamics simulations of molecule **1** in COX-2 binding site. (a) Snapshots 3D (A) from stable segments of molecular dynamics simulations show that **1** bound to Arg120, Tyr 355, and Arg 513 by three h-bonds and one salt bridge. (B) The interactions occur more than 30.0% of the simulation time in the selected trajectory (0.00 through 50.05 nsec). (b) The interaction diagram demonstrates the percentage interaction of ligand **1** with surrounding residues. (c) Root mean-squared fluctuation (RMSF) of molecule **1** fitted on the protein (red) or ligand (purple line). The atom numbers of **1** (A) correspond to the RMSF plot *x*-axis (B). The ligand is shown in green ball-and-stick, oxygen atoms in red, carbon atoms in black, and nitrogen atoms in blue.

Simulation time 50 ns with RMSD protein and ligand RMSD in the range 1-3 Å showed that the system had equilibrium. From the early stages of the simulation process, the h-bond between ligand **1** and Ser 530 in docking pose was not stable, whereby the Arg 120, Tyr 355, and Arg 513 were the main contact residues over the course of the trajectory. Arg 120 stands out with a percentage of h-bond interactions greater than 1.2, followed by Tyr 355 at 1.1 and Arg 513 at 0.7. The contribution of water bridges was minor for Arg 120 and Tyr 355 but 0.5 for Arg 513 Figures 6(a), B and 6(b). On the other hand, quantifying the RMSF showed that most atoms ligand **1** fluctuated in the range of 1.5 when fitted with COX-2 protein. This finding demonstrated the thermodynamic stability of the ligand-protein system (Figure 6(c)). In general, the *in silico* research exhibited the COX-2 inhibitor capacity of compound **1** by generating h-bonds at the positions of Arg 120, Tyr 355, and Arg 513 residues.

## 4. Discussion

Compounds **1-3** were isolated from *n*-hexane extract of *D. benenica* Q.B.Nguyen & Škorničk rhizome. The anti-inflammatory and antimicrobial bioactivities of **1-3** were evaluated *in vivo* and *in vitro,* respectively, revealing that 1 inhibited inflammation, while **1** and **2** had antimicrobial activity against Gram-positive bacteria.

Since inflammation is involved in diseases, the finding of compounds that inhibit inflammation is of interest. Many methods have been applied to determine the anti-inflammation activity. Carrageenan-induced paw edema is one of the *in vivo* models which has been used to evaluate anti-inflammatory activities and has long been established as a valid model to study new anti-inflammatory drugs. Carrageenan causes acute inflammation by inhibiting monocyte migration and preventing prostaglandin release [40, 41]. Diclofenac is a commonly nonsteroidal anti-inflammatory drug (NSAID) that is effective for treating acute, chronic pain and inflammatory conditions. Similar to other NSAIDs, diclofenac inhibits prostaglandin release by targeting cyclooxygenase-1 and cyclooxygenase-2 (COX-1 and COX-2) [42]. Here, **1** significantly alleviated the edema caused by carrageenan in mice comparably to diclofenac (Figures 3 and 4). Our result is consistent with the anti-inflammatory effect of *p-*coumaric acid, as *p-*coumaric acid isolated from *Oldenlandia diffusa* also ameliorated inflammation symptoms [23]. In arthritic rats, *p-*coumaric acid prevented the expression of tumor necrosis factor (TNF-*α*) in synovial tissues and circulating immune complexes [43]. In lipopolysaccharide- (LPS-) stimulated RAW264.7, *p-*coumaric acid significantly inhibited the expression of inducible nitric oxide synthase, COX-2, interleukin-1*β*, and TNF-*α*. Here, for the first time, we demonstrated the anti-inflammatory properties of *trans-o*-coumaric acid (Figures 2 and 3). Liao et al. found that *trans*-cinnamic did not have anti-inflammatory activity [44]. In our study, *trans-*cinnamic acid exhibited low anti-inflammatory activity (Figure 2). However, our result for **3** differed from that reported for borneol, which significantly decreased the expression of inflammatory factors, including nitric oxide, TNF-*α*, and interleukin-6 in LPS-induced RAW 264.7 macrophages *in vitro* [29]. In another study, mice treated with ≥50 mg/kg borneol showed significantly reduced migration of carrageenan-induced leukocytes to the peritoneal cavity [30]. In our study, 20 mg/kg borneol slightly ameliorated the swelling compared to the negative control, by approximately 38% after 4 h of carrageenan treatment (Figure 3).

One study revealed information on the growth inhibition of *trans-o*-coumaric acid against *B. subtilis*, *Proteus vulgaris*, and *S. aureus* [26], whereas another study showed the antiproliferation activity of *p*-coumaric acid against Gram-positive (*Streptococcus pneumoniae*, *S. aureus*, and *B. subtilis*) and Gram-negative (*E. coli*, *Shigella dysenteriae*, and *Salmonella typhimurium*) bacteria with MICs of 10-80 *μ*g/mL [45]. Taofiq et al. demonstrated the antimicrobial effects of *p-*coumaric acid against Gram-positive (methicillin-resistant and methicillin-sensitive *S. aureus* and *Enterococcus faecalis*) and Gram-negative (*E. coli* and *Proteus mirabilis*) with MICs of 0.5-1 and 2.5 mg/mL, respectively [20]. Similarly, a nanoemulsion of *trans*-cinnamic acid reduced the growth of *S. aureus*, *S. typhimurium*, and *P. aeruginosa* with MICs of 0.78, 1.56, and 3.13 mg/mL, respectively [46]. *Trans*-cinnamic acid prevented the proliferation of Gram-positive strains (*B. subtilis*, *Bacillus thuringiensis*, and *Lysinibacillus xylanilyticus*) with a MIC of 1 mg/mL and Gram-negative strains (*E. coli*, *Klebsiella pneumoniae*) with a MIC of >1 mg/mL. *Trans*-cinnamic acid was more effective against *Aeromonas sobria* (MIC = 0.25 mg/mL) [47]. Cinnamic acid displayed comparable activity against Gram-positive (methicillin-resistant and methicillin-sensitive *S. aureus* and *E. faecalis*) and Gram-negative (*E. coli* and *Proteus mirabilis*) strains with a MIC of ~1 mg/mL [20]. Borneol had antimicrobial effects against Gram-positive bacteria such as *S. aureus* and *E. faecalis* (MIC = 2 − 4 *μ*L/mL) [27]. In another study, however, borneol lacked antimicrobial activity [48]. In our study, **1** and **2** reduced the proliferation of Gram-positive strains (MICs = 1.52-3.37 mM) more effectively than **3** (MIC = 6.49 mM) (Table 1). **1-3** had low antimicrobial activity against Gram-negative bacterial strains (*E. coli* and *P. aeruginosa*) (MICs = 6.1-6.75 mM).

## 5. Conclusions

We isolated **1-3** from an *n*-hexane extract of *D. benenica* Q.B.Nguyen & Škorničk rhizomes and evaluated their anti-inflammatory and antimicrobial bioactivities. In a carrageenan-induced paw edema assay, **1** inhibited the intensity of edema comparably to diclofenac. The *in silico* research exhibited that the anti-inflammation activity of **1** may be mediated by inhibiting COX-2 via generating h-bonds at the positions of Arg 120, Tyr 355, and Arg 513 residues. Furthermore, in an antimicrobial assay, **1** and **2** hampered the growth of Gram-positive (*S. aureus* and *B. subtilis*) but had low activity against Gram-negative strains. Taken together, our results revealed that **1** isolated from *D. benenica* might have the anti-inflammatory function whereas **1** and **2** could reduce the growth of several Gram-positive bacteria.

## Figures and Tables

**Table 1:** The MICs of **1-3** on different bacterial strains.

Bacterial strains	MICs of compounds (mM)			
	Ciprofloxacin	**1**	**2**	**3**
*Staphylococcus aureus*	0.648 × 10^−3^	1.52	1.69	6.49
*Bacillus subtilis*	0.648 × 10^−3^	3.04	3.37	6.49
*Pseudomonas aeruginosa*	0.041 × 10^−3^	6.10	6.75	6.49
*Escherichia coli*	0.041 × 10^−3^	6.1	6.75	6.49

**Table 2:** The XP docking scores and MM-GBSA binding free energy estimations (kcal/mol) of three molecules **1**, **2**, and diclofenac reference drug.

Compounds	COX-1				COX-2			
	XP GlideScore (kcal/Mol)	MM-GBSA (kcal/Mol)	No of h-bonds	Residues	XP GlideScore (kcal/Mol)	MM-GBSA (kcal/Mol)	No of h-bonds	Residues
**1**	-5.7	-8.2	2	Ser516, Phe518	-6.5	-29.7	3	Arg120, Tyr355, Ser530
**2**	-5.1	-8.8	2	Ser516, Phe518	-5.3	-25.2	2	Arg120, Tyr355
Diclofenac	-7.5	-29.3	—	—	-8.5	-33.9	1	Ser 530

## Data Availability

The data used to support the findings of this study are available from the corresponding author upon request.

## Supplementary Materials

Supplementary Materials Figure S1: FT-IR spectrum (KBr) of compound (**1**). Figure S2: ^1^H-NMR spectrum (MeOD, 500 MHz) of compound (**1**). Figure S3: ^1^H-NMR spectrum (MeOD, 500 MHz) of compound (**1**) (expansion 1). Figure S4: ^13^C-NMR spectrum (MeOD, 125 MHz) compound (**1**). Figure S5: ^13^C-NMR spectrum (MeOD, 125 MHz) compound (**1**) (expansion 1). Figure S6: DEPT spectrum (MeOD, 125 MHz) compound (**1**) (expansion 1). Figure S7: HRESIMS spectrum of compound (**1**). Figure S8: FT-IR spectrum (KBr) of compound (**2**). Figure S9: ^1^H-NMR spectrum (MeOD, 500 MHz) of compound (**2**). Figure S10: ^1^H-NMR spectrum (MeOD, 500 MHz) of compound (**2**) (expansion 1). Figure S11: ^13^C-NMR spectrum (MeOD, 125 MHz) compound (**2**). Figure S12: ^13^C-NMR spectrum (MeOD, 125 MHz) compound (**2**) (expansion 1). Figure S13:. DEPT spectrum (MeOD, 125 MHz) compound (**2**). Figure S14: HRESIMS spectrum of compound (**3**). Figure S15: FT-IR spectrum (KBr) of compound (**3**). Figure S16: ^1^H-NMR spectrum (CDCl_3_, 500 MHz) of compound (**3**). Figure S17: ^1^H-NMR spectrum (CDCl_3_, 500 MHz) of compound (**3**) (expansion 1). Figure S18: ^1^H-NMR spectrum (CDCl_3_, 500 MHz) of compound (**3**) (expansion 2). Figure S19: ^13^C-NMR spectrum (CDCl_3_, 125 MHz) compound (**3**). Figure S20: ^13^C-NMR spectrum (CDCl_3_, 125 MHz) compound (**3**) (expansion 1). Figure S21: DEPT spectrum (CDCl_3_, 125 MHz) compound (**3**). Figure S22: HRESIMS spectrum of compound (**3**). Figure S23: Binding poses of three molecule 1, 2 and the diclofenac with COX-1 proteins.
