# Supplementary material for: Anti-Inflammatory and Antimicrobial Activities of Compounds Isolated from Distichochlamys benenica
Source: Biomed Res Int. 2021 Apr 6;2021:6624347. doi: 10.1155/2021/6624347 (PMC8046538; doi:10.1155/2021/6624347)

**SUPPLEMENTARY MATERIAL**

**Anti-inflammatory and anti-microbial activities of compounds isolated from *Distichochlamys benenica***

Ty Viet Pham^1^, Hanh Nhu Thi Hoang^2^, Hoai Thi Nguyen^3^, Hien Minh Nguyen^4^, Cong Thang Huynh^4^, Thien Y Vu^4^, Anh Thu Do^5^, Hoai Nguyen Nguyen^6^, Bich Hang Do^4,*^

1. *Faculty of Chemistry, University of Education, Hue University, 34 Le Loi, Hue City, Vietnam;*
2. *Faculty of Engineering and Food Technology, Hue University of Agriculture and Forestry, Hue University, 102 Phung Hung, Hue City, Viet Nam;*
3. *Faculty of Pharmacy, University of Medicine and Pharmacy, Hue University, 06 Ngo Quyen, Hue City, Vietnam;*
4. *Faculty of Pharmacy, Ton Duc Thang University, Ho Chi Minh City, Vietnam*
5. *English Faculty, Foreign Trade University - Ho Chi Minh City Campus, Vietnam*
6. *Faculty of Biotechnology, Ho Chi Minh City Open University, Ho Chi Minh City, Vietnam*

** Corresponding author: Bich Hang Do*

*Address: Faculty of Pharmacy, Ton Duc Thang University, Ho Chi Minh City, Vietnam*

*Email.* [*dobichhang@tdtu.edu.vn*](mailto:dobichhang@tdtu.edu.vn)

**Abstract**

*Distichochlamys* *benenica* is a native black ginger that grows in Vietnam. In point of fact, there is limitation of available information in the literature making mention of the chemical constituents and bioactive properties of this plant. This study aims to isolate *trans-o*-coumaric acid (**1**), *trans*-cinnamic acid (**2**), and borneol (**3**) from the rhizomes of *D. benenica* Q.B.Nguyen & Škorničk and evaluate the anti-inflammatory and anti-microbial activities of **1**-**3** using the carrageenan paw oedema model and the dilution broth method, respectively. This revealed that **1** was as effective as diclofenac in reducing the intensity of the oedema development. The *in-silico* research showed that the activity of **1** might derive from inhibiting COX-2 by generating h-bonds at the positions of Arg 120, Tyr 355, and Arg 513 residues. The antimicrobial activities of against Gram-positive strains (*Staphylococcus aureus* and *Bacillus subtilis*) were comparable, with the minimum inhibitory concentrations ranging from 1.52-3.37 mM. This is the first study of the bioactivity of compounds isolated from *D. benenica Q.B.Nguyen* & Škorničk. Our results suggest that **1** may be a nature-derived compound which demonstrates the anti-inflammatory properties and inhibit the proliferation of several Gram-positive bacteria.

Keywords: *trans-o*-coumaric acid, *trans*-cinnamic acid, borneol, inflammation, antimicrobial activity, *Distichochlamys benenica*.

List of Contents

Fig. S1 FT-IR spectrum (KBr) of compound (1)

**Fig. S2** ^1^H-NMR spectrum (MeOD, 500 MHz) of compound (**1**)

**Fig. S3** ^1^H-NMR spectrum (MeOD, 500 MHz) of compound (**1**) (expansion 1)

**Fig. S4** ^13^C**-**NMR spectrum (MeOD, 125 MHz) compound (**1**)

**Fig. S5** ^13^C**-**NMR spectrum (MeOD, 125 MHz) compound (**1**) (expansion 1)

**Fig. S6** DEPT spectrum (MeOD, 125 MHz) compound (**1**) (expansion 1)

**Fig. S7** HRESIMS spectrum of compound (**1**)

Fig. S8 FT-IR spectrum (KBr) of compound (2)

**Fig. S9** ^1^H-NMR spectrum (MeOD, 500 MHz) of compound (**2**)

**Fig. S10** ^1^H-NMR spectrum (MeOD, 500 MHz) of compound (**2**) (expansion 1)

**Fig. S11** ^13^C**-**NMR spectrum (MeOD, 125 MHz) compound (**2**)

**Fig. S12** ^13^C**-**NMR spectrum (MeOD, 125 MHz) compound (**2**) (expansion 1)

**Fig. S13** DEPT spectrum (MeOD, 125 MHz) compound (**2**)

**Fig. S14** HRESIMS spectrum of compound (**3**)

Fig. S15 FT-IR spectrum (KBr) of compound (3)

**Fig. S16** ^1^H-NMR spectrum (CDCl_3_, 500 MHz) of compound (**3**)

**Fig. S17** ^1^H-NMR spectrum (CDCl_3_, 500 MHz) of compound (**3**) (expansion 1)

**Fig. S18** ^1^H-NMR spectrum (CDCl_3_, 500 MHz) of compound (**3**) (expansion 2)

**Fig. S19** ^13^C**-**NMR spectrum (CDCl_3_, 125 MHz) compound (**3**)

**Fig. S20** ^13^C**-**NMR spectrum (CDCl_3_, 125 MHz) compound (**3**) (expansion 1)

**Fig. S21** DEPT spectrum (CDCl_3_, 125 MHz) compound (**3**)

**Fig. S22** HRESIMS spectrum of compound (**3**)

**Fig. S23** Binding poses of three molecule **1**, **2** and the diclofenac with COX-1 proteins.


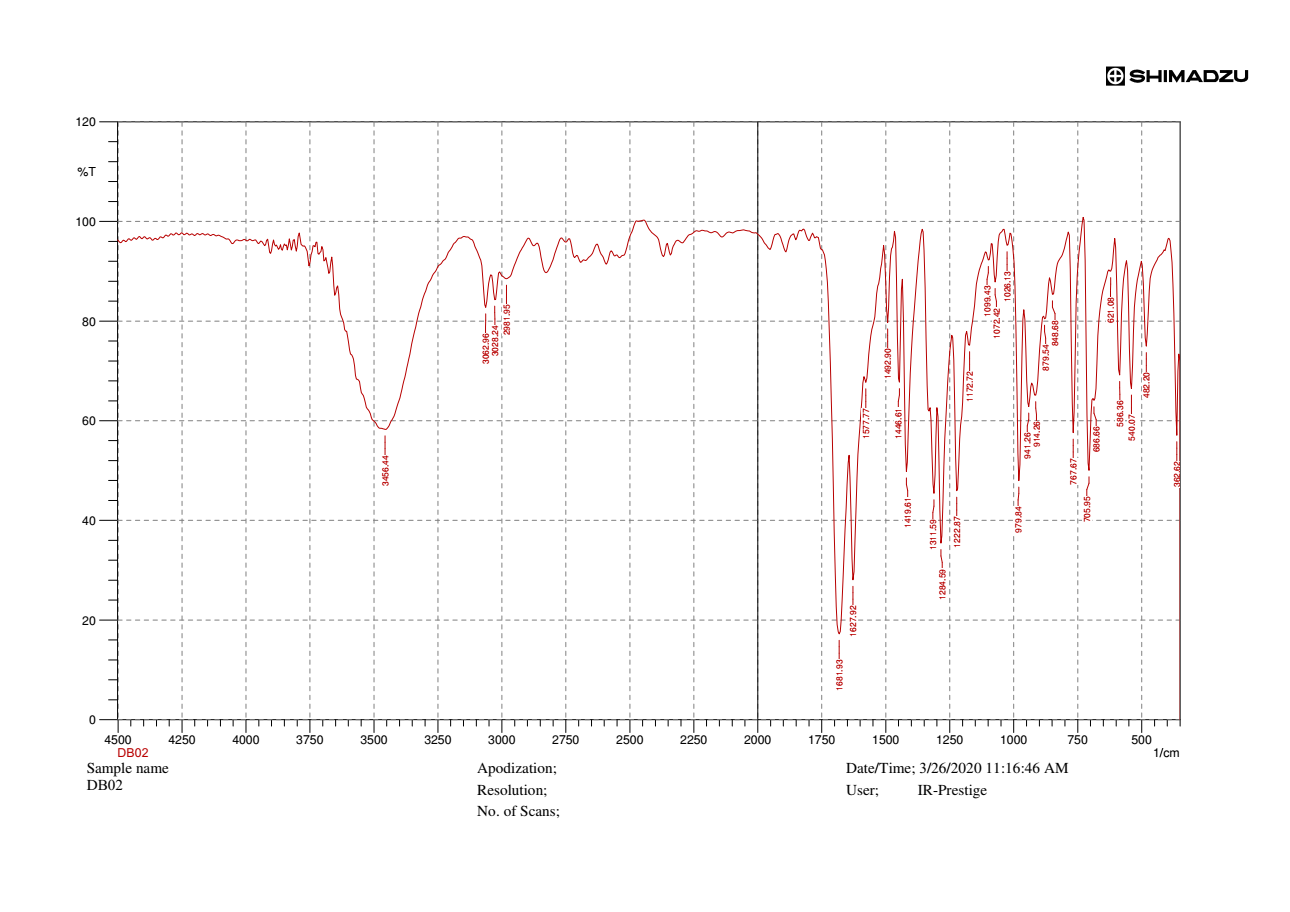


Fig. S1 FT-IR spectrum (KBr) of compound (1)


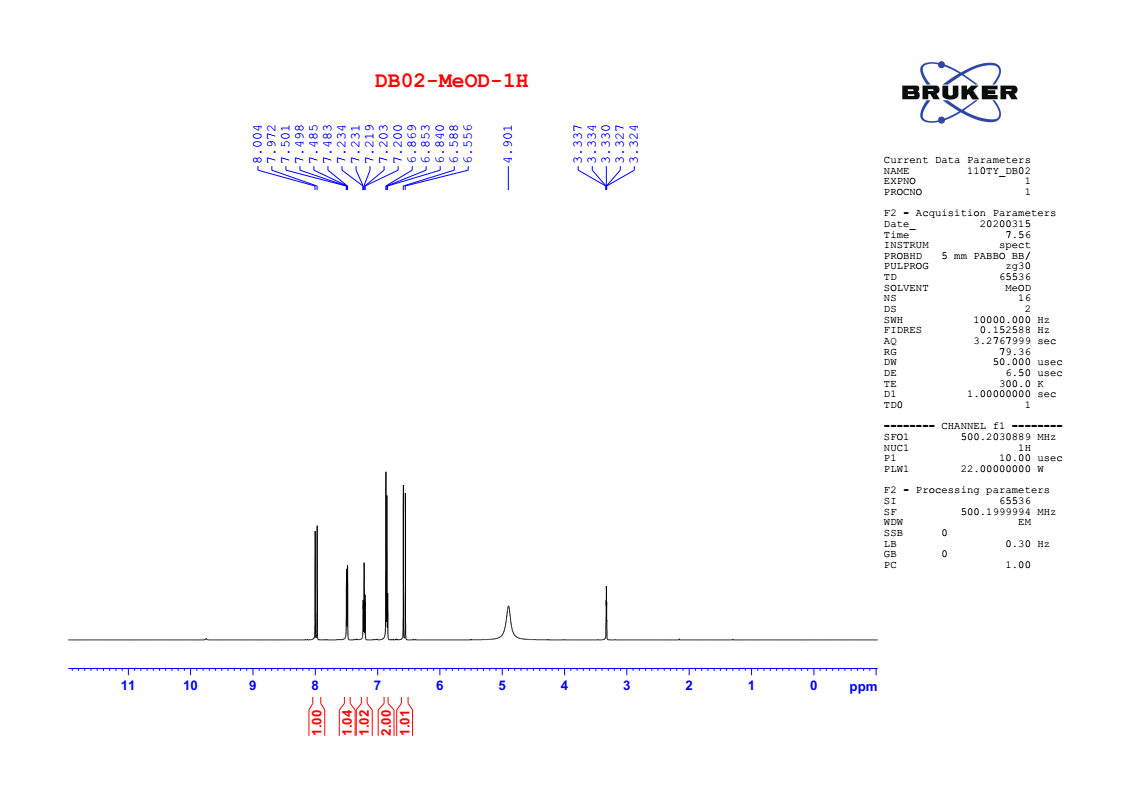


**Fig. S2** ^1^H-NMR spectrum (MeOD, 500 MHz) of compound (**1**)

**
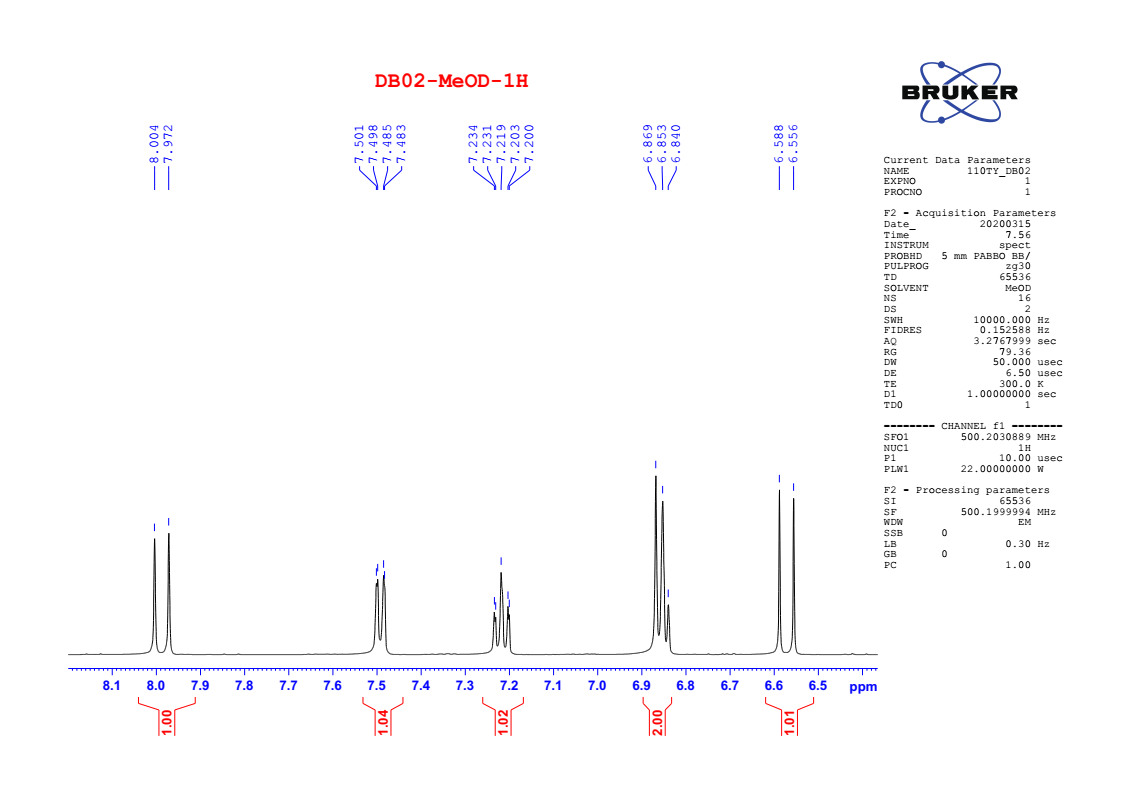
**

**Fig. S3** ^1^H-NMR spectrum (MeOD, 500 MHz) of compound (**1**) (expansion 1)


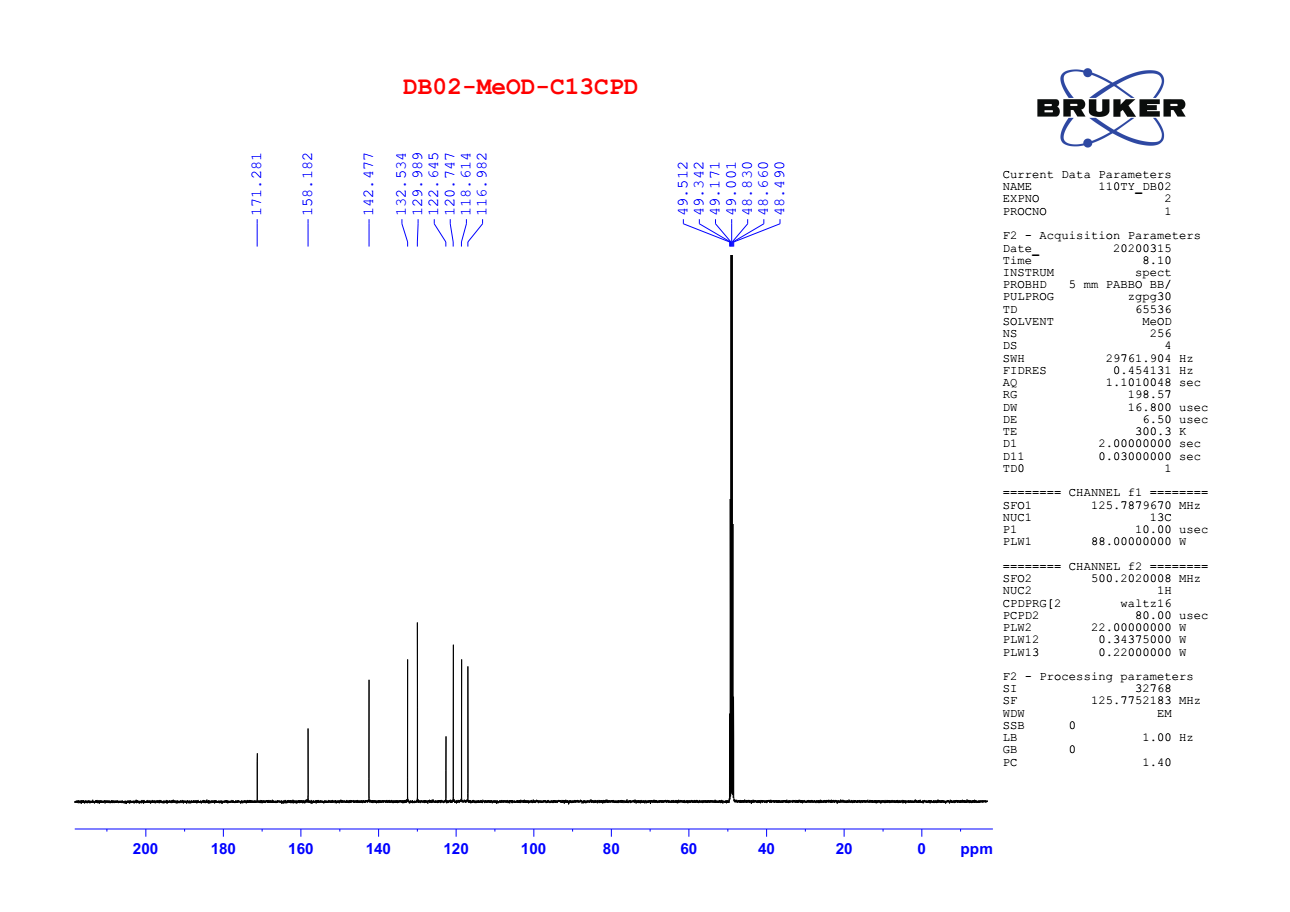


**Fig. S4** ^13^C**-**NMR spectrum (MeOD, 125 MHz) compound (**1**)


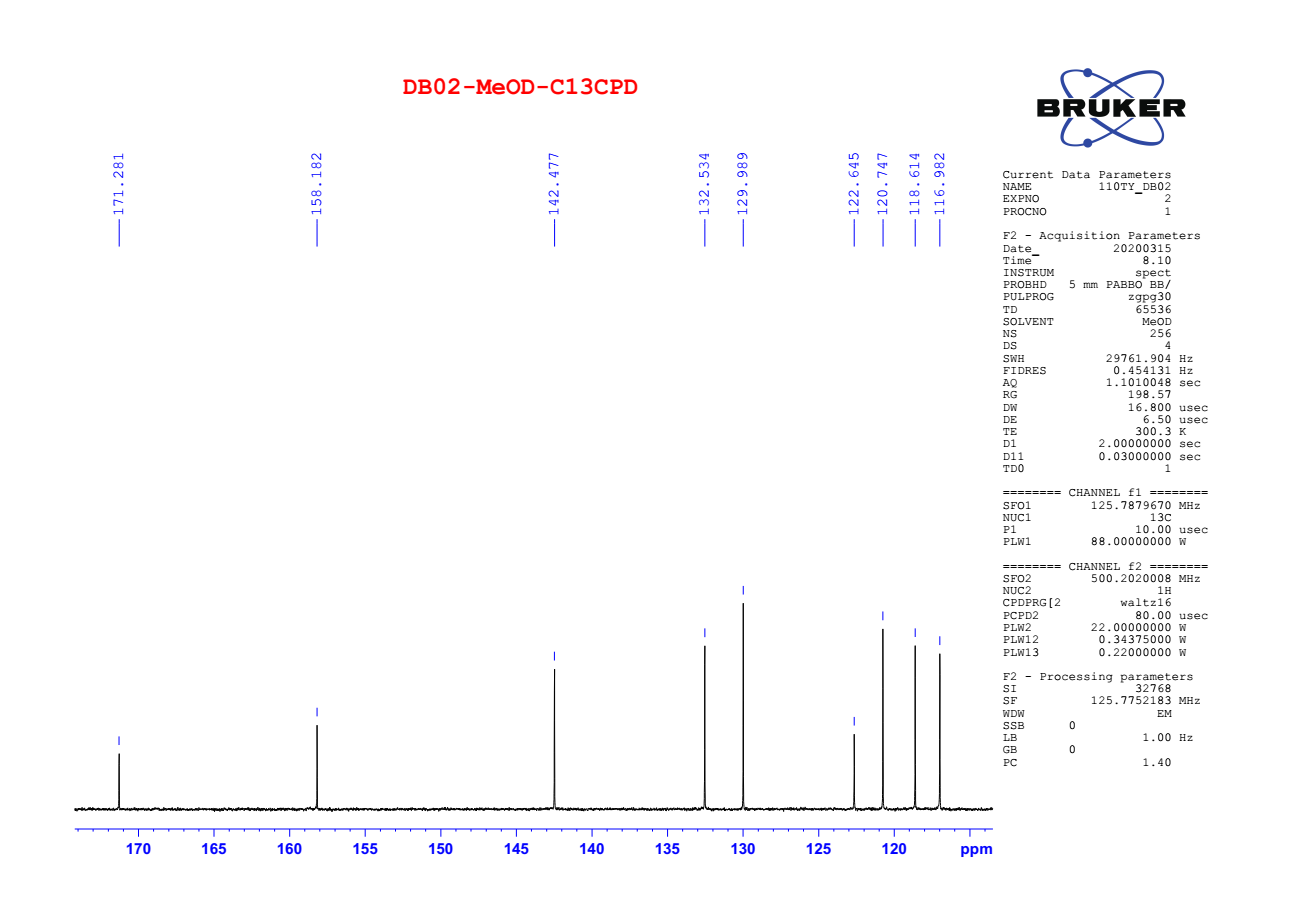


**Fig. S5** ^13^C**-**NMR spectrum (MeOD, 125 MHz) compound (**1**) (expansion 1)

**
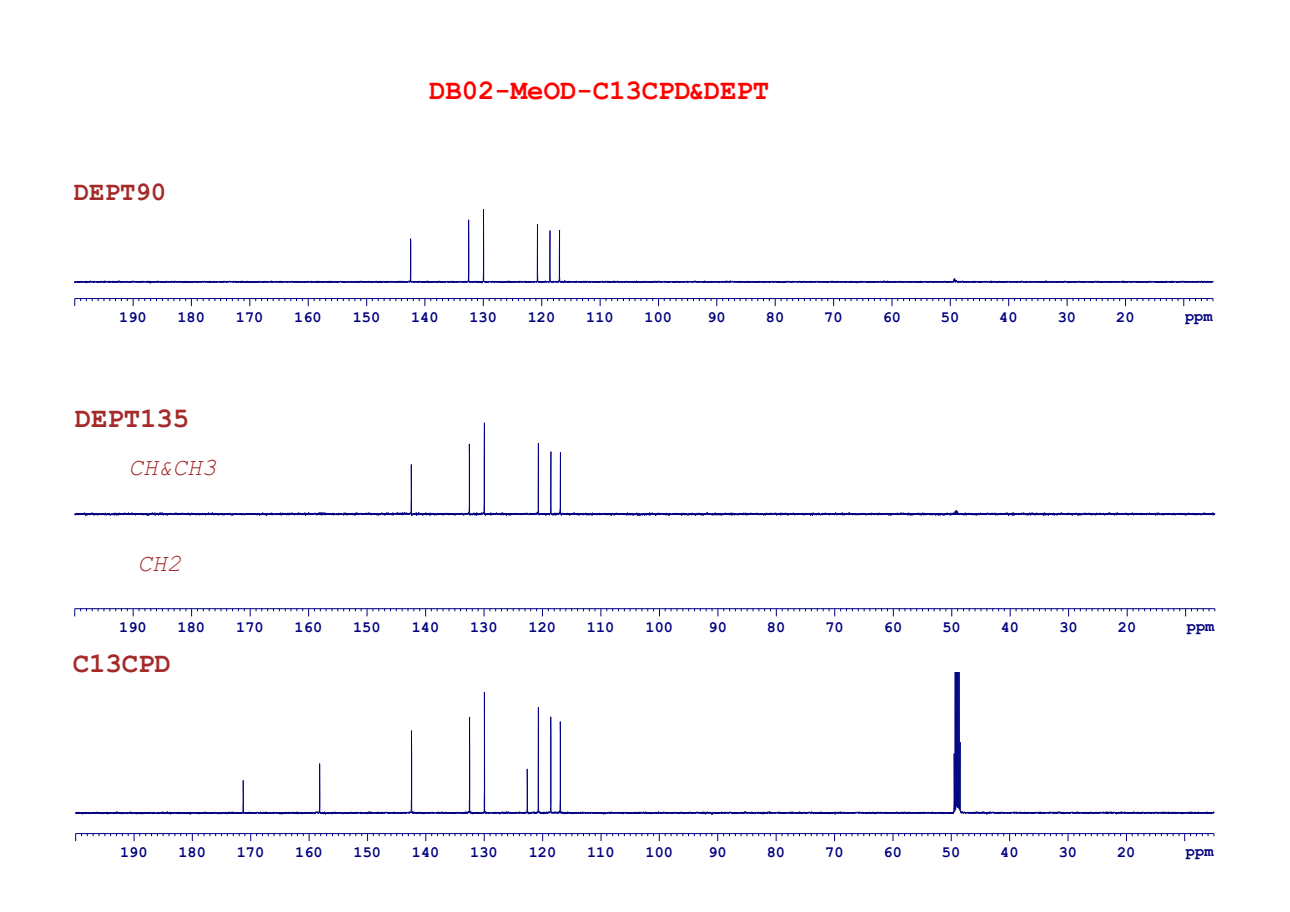
**

**Fig. S6** DEPT spectrum (MeOD, 125 MHz) compound (**1**) (expansion 1)

**
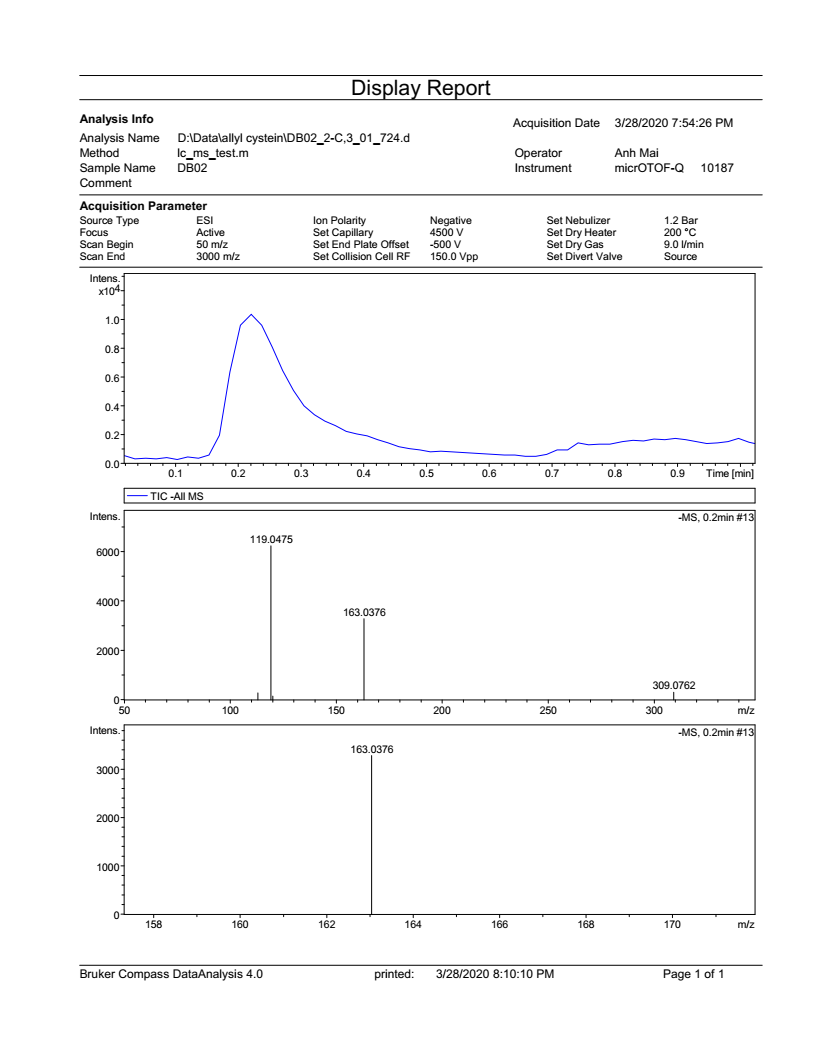
**

**Fig. S7** HRESIMS spectrum of compound (**1**)


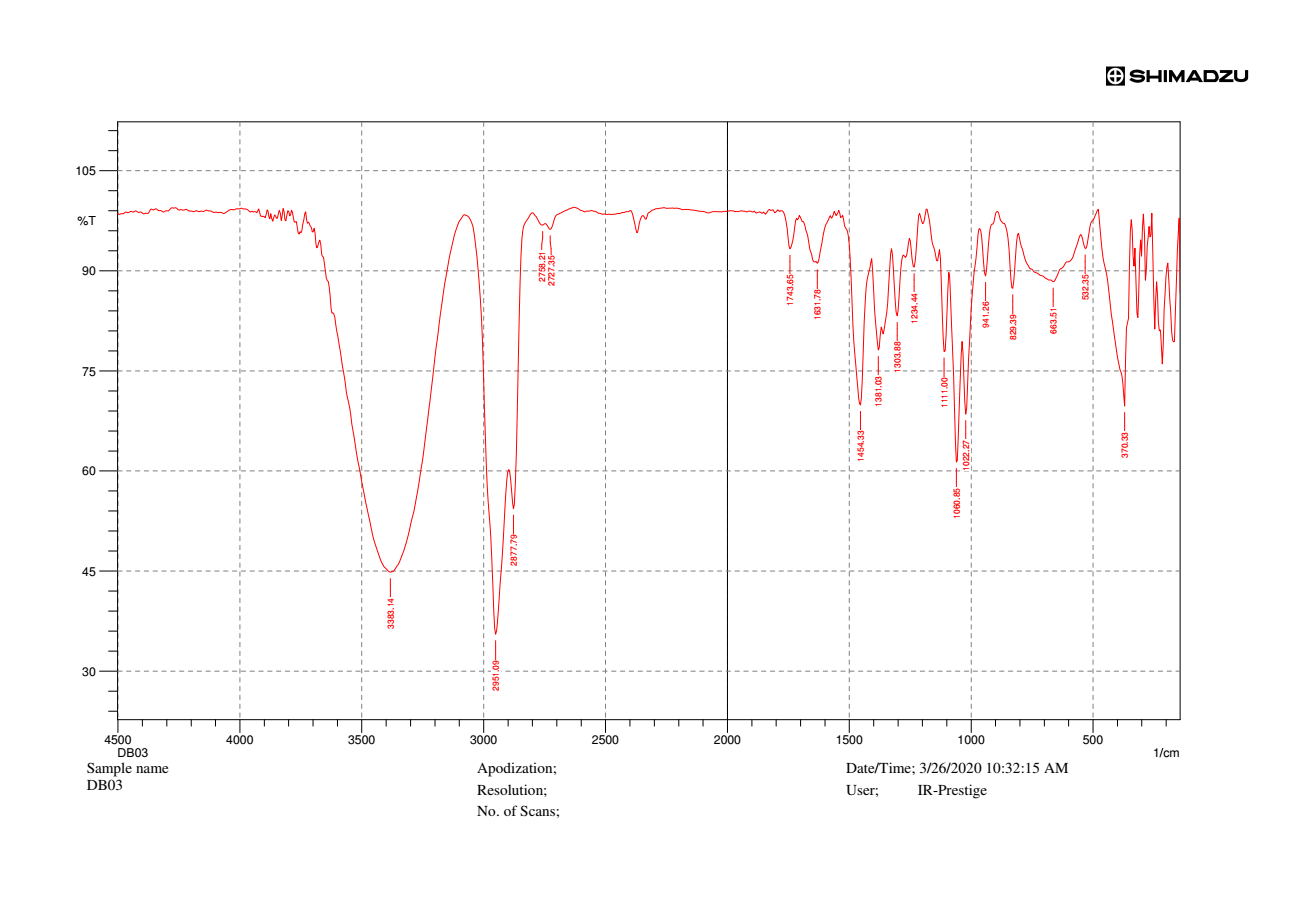


Fig. S8 FT-IR spectrum (KBr) of compound (2)

**
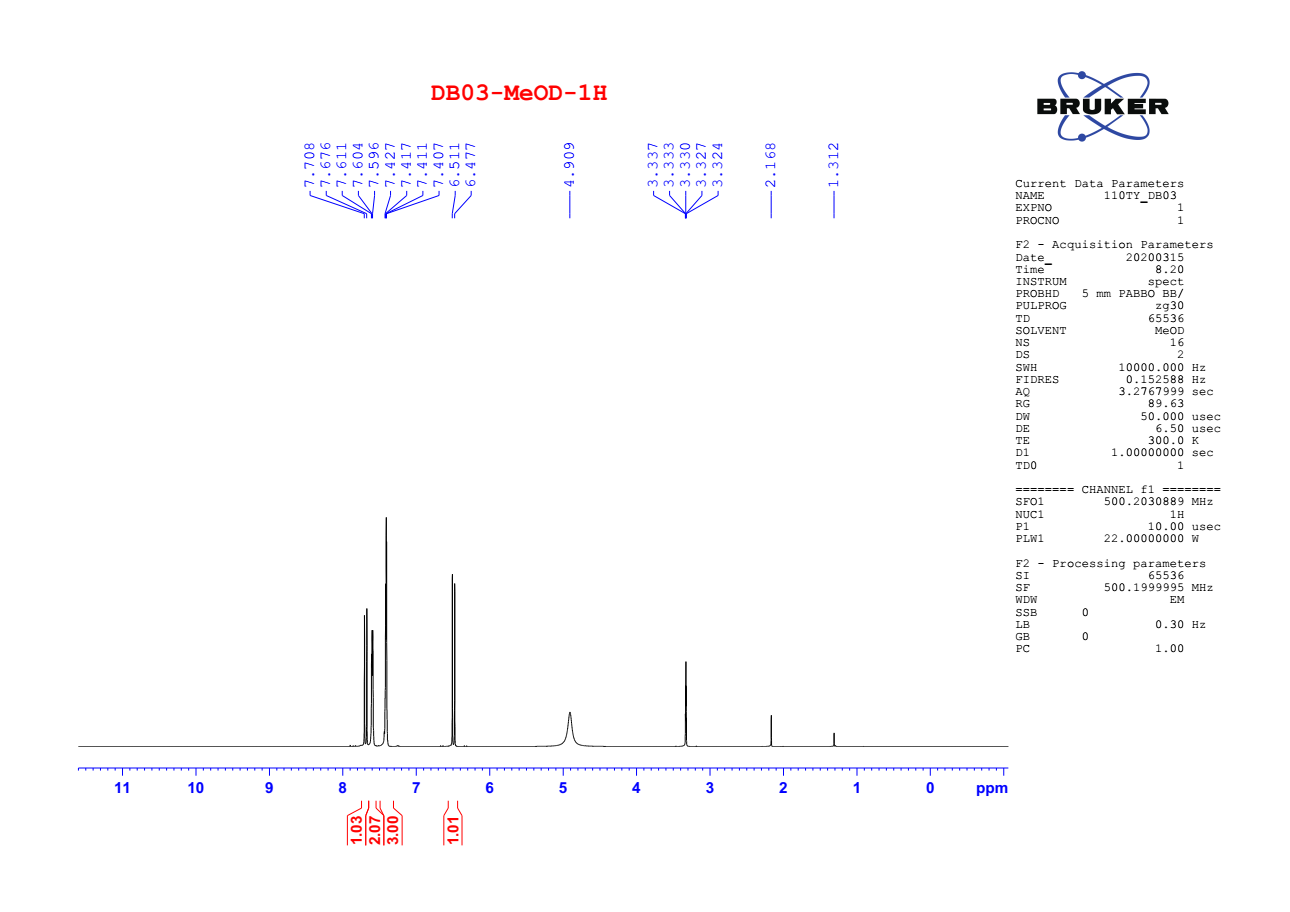
**

**Fig. S9** ^1^H-NMR spectrum (MeOD, 500 MHz) of compound (**2**)

**
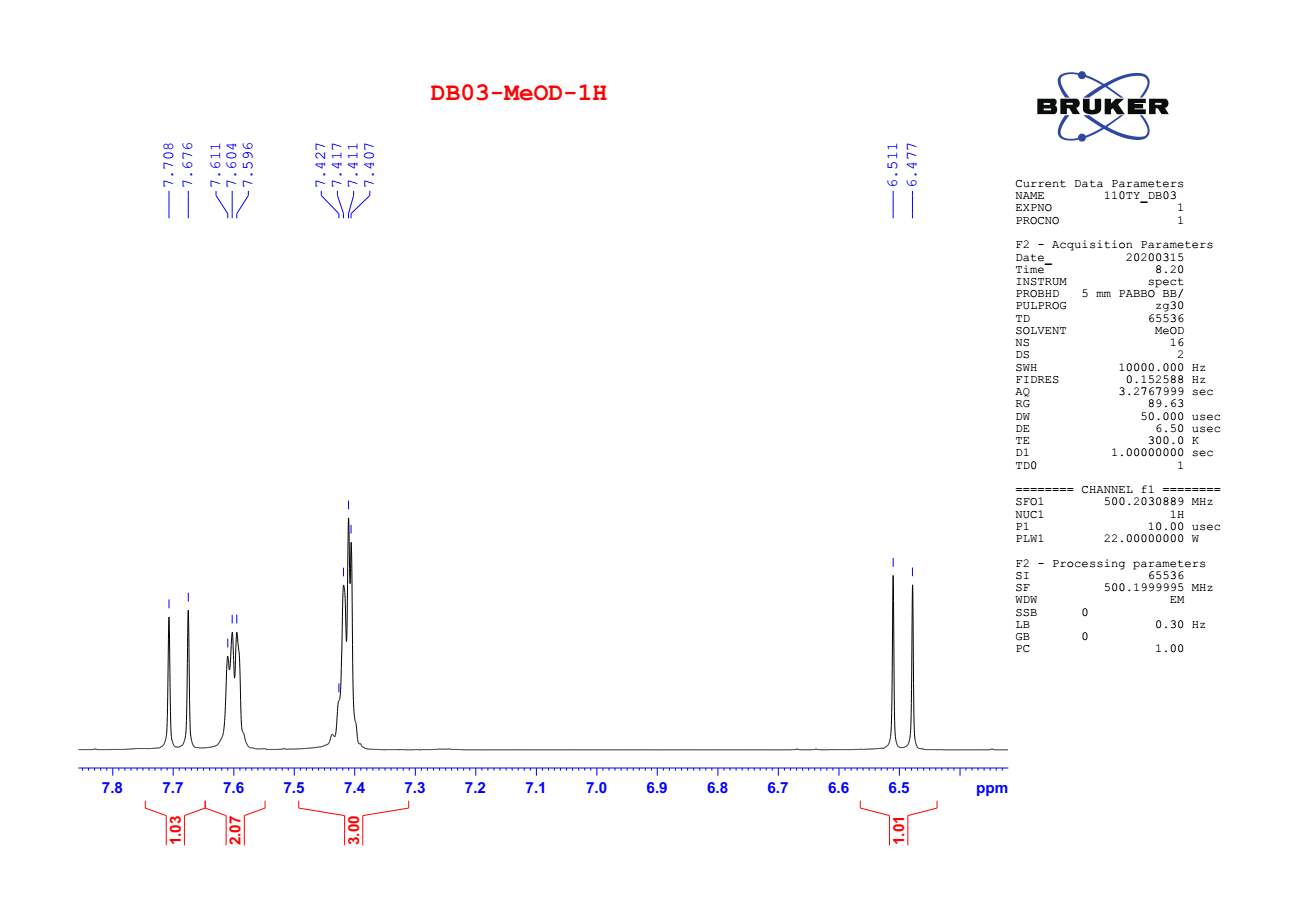
**

**Fig. S10** ^1^H-NMR spectrum (MeOD, 500 MHz) of compound (**2**) (expansion 1)

**
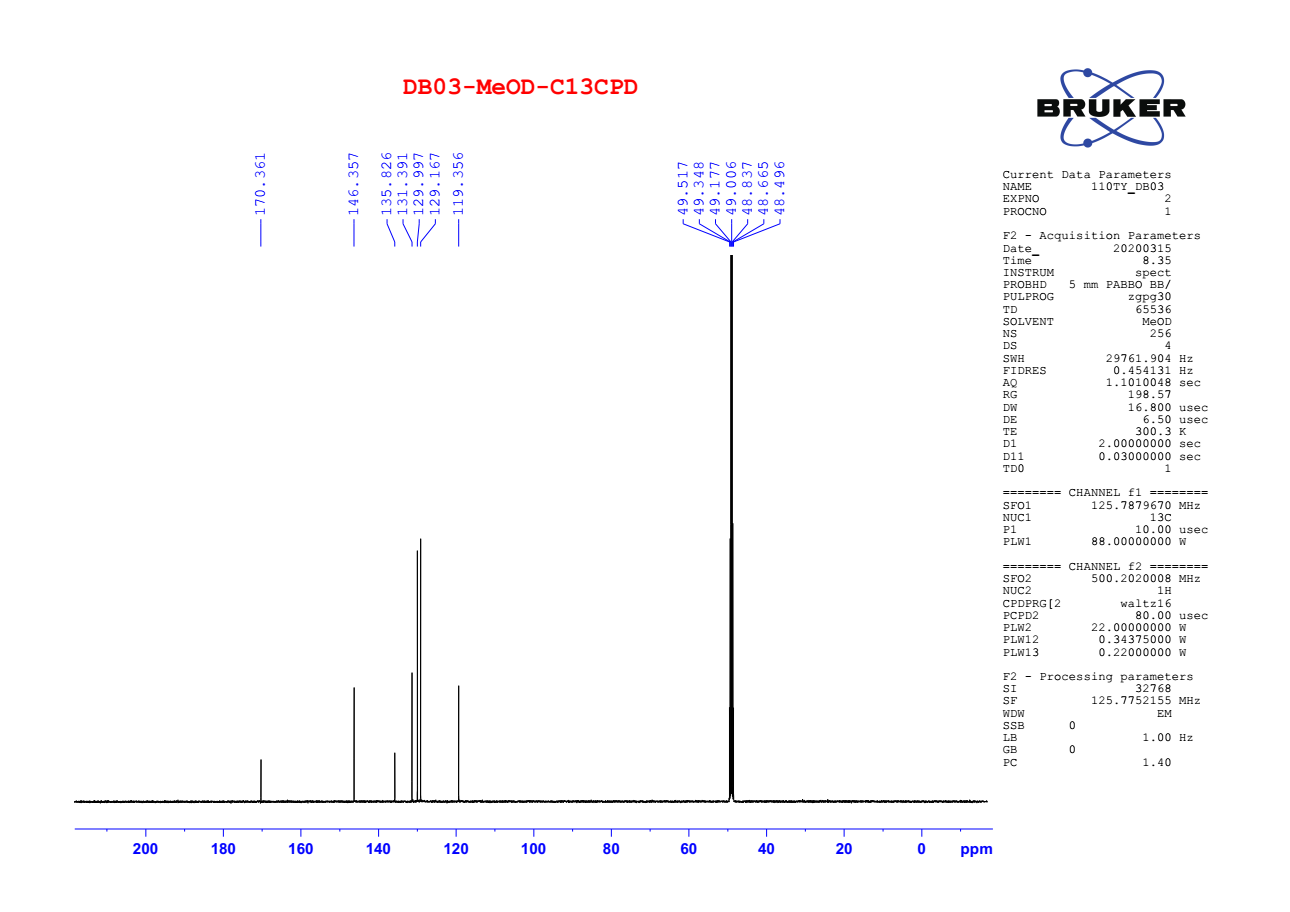
**

**Fig. S11** ^13^C**-**NMR spectrum (MeOD, 125 MHz) compound (**2**)

**
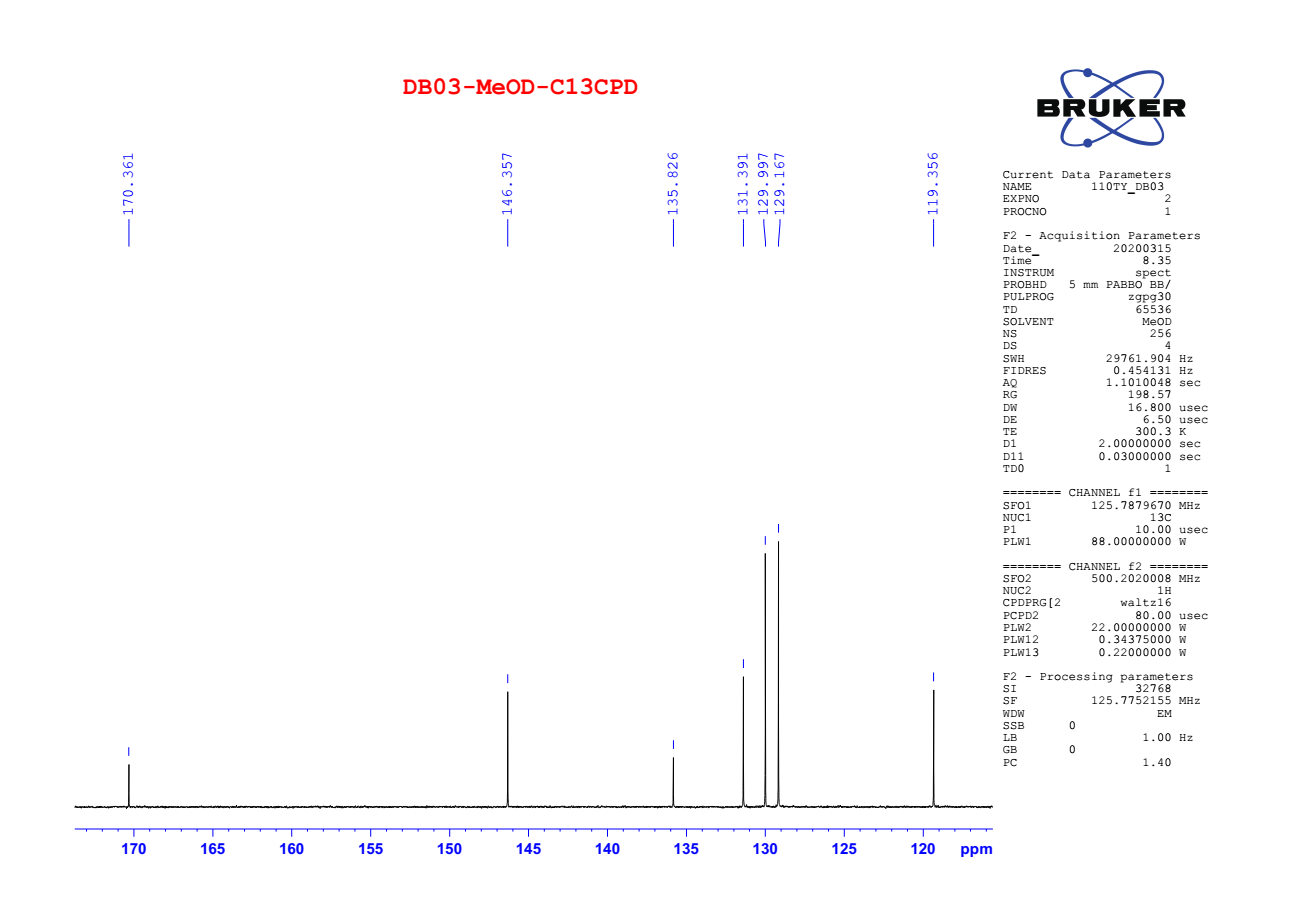
**

**Fig. S12** ^13^C**-**NMR spectrum (MeOD, 125 MHz) compound (**2**) (expansion 1)

**
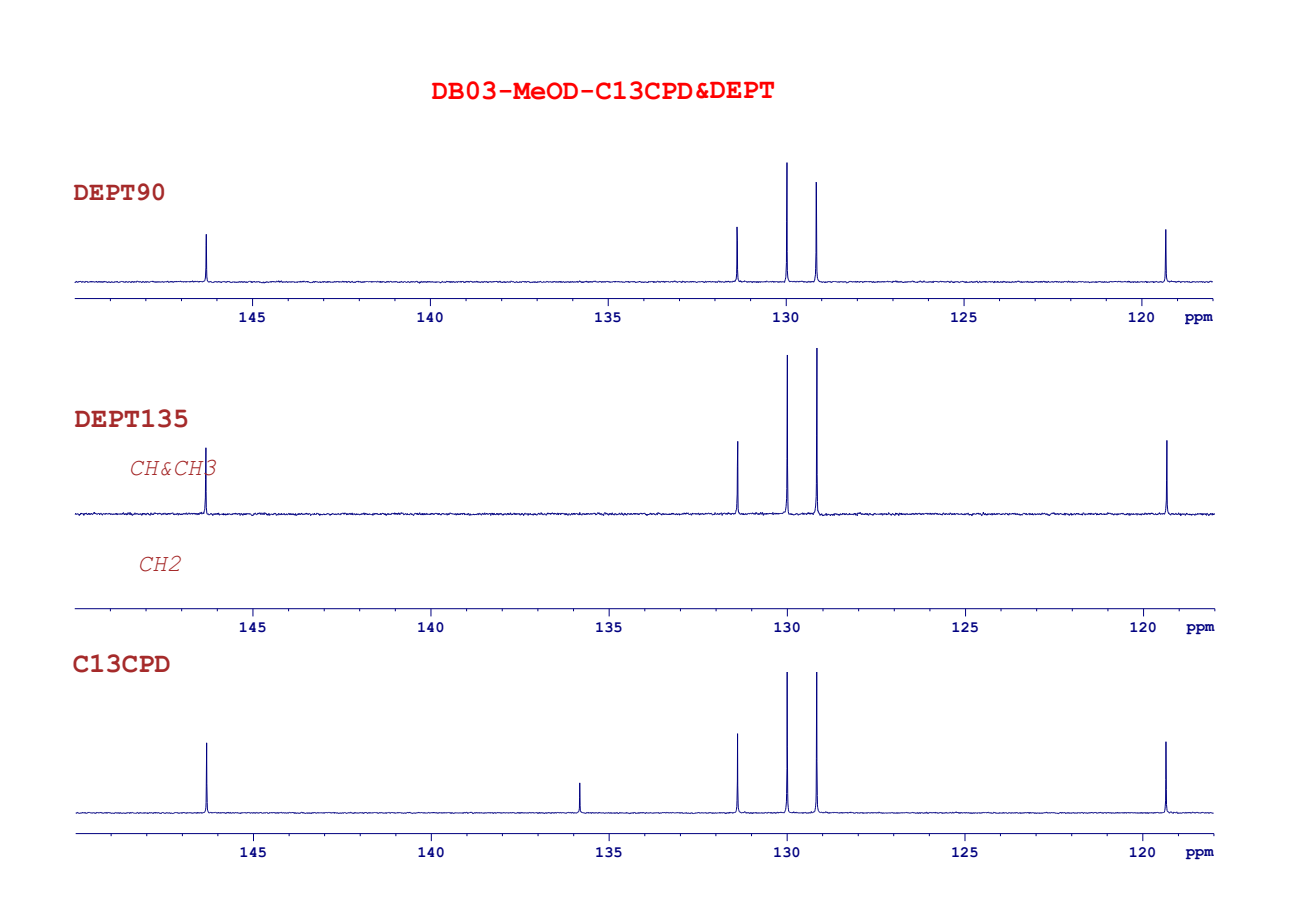
**

**Fig. S13** DEPT spectrum (MeOD, 125 MHz) compound (**2**)

**
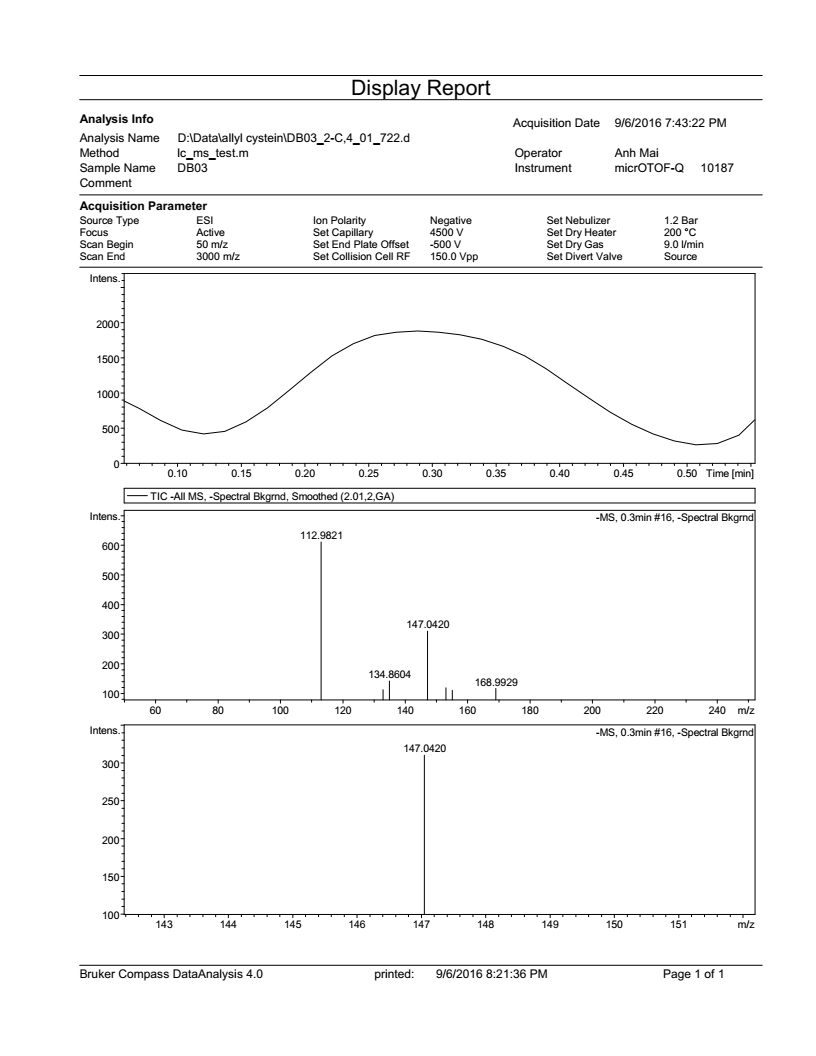
**

**Fig. S14** HRESIMS spectrum of compound (**3**)


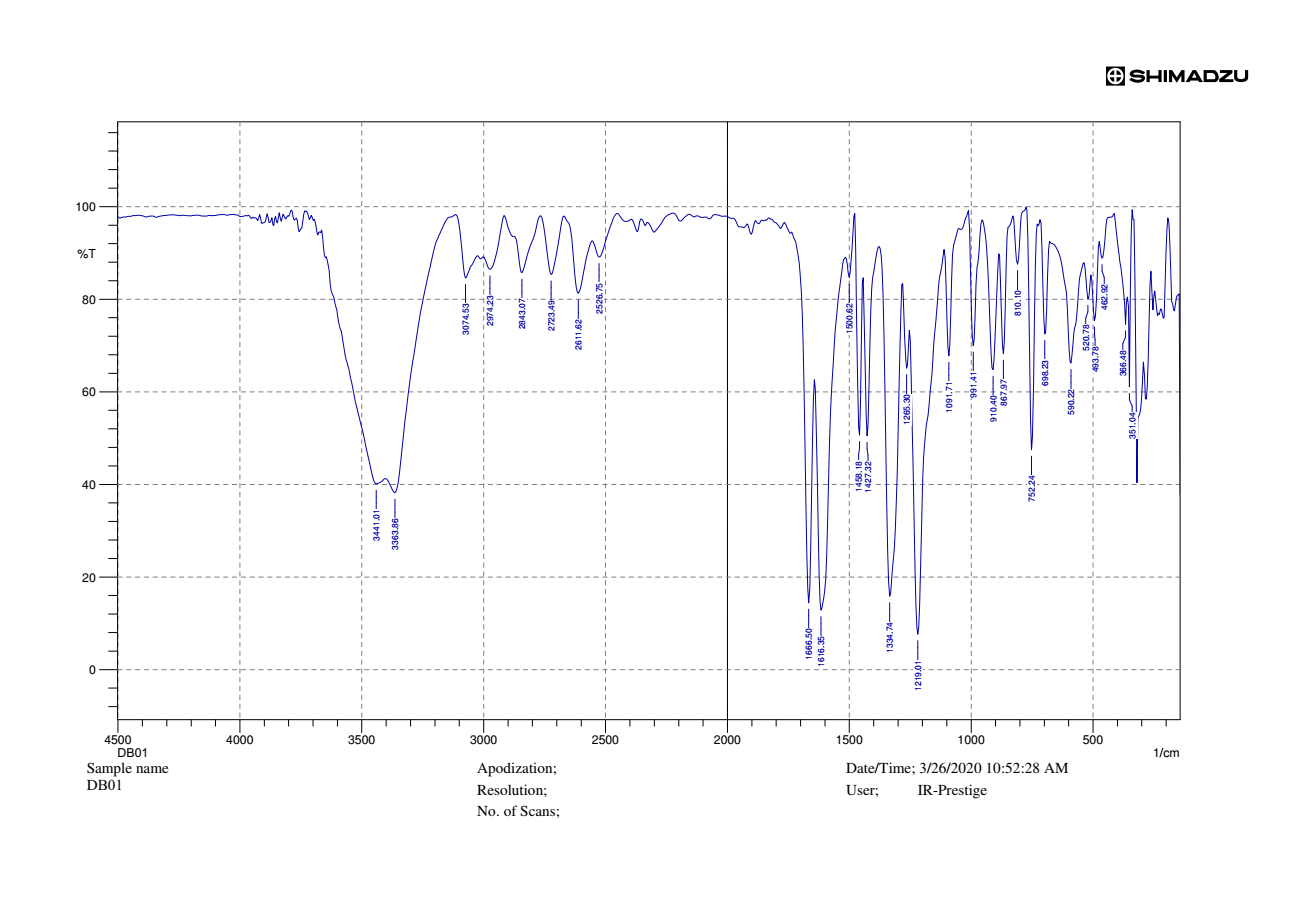


Fig. S15 FT-IR spectrum (KBr) of compound (3)

**
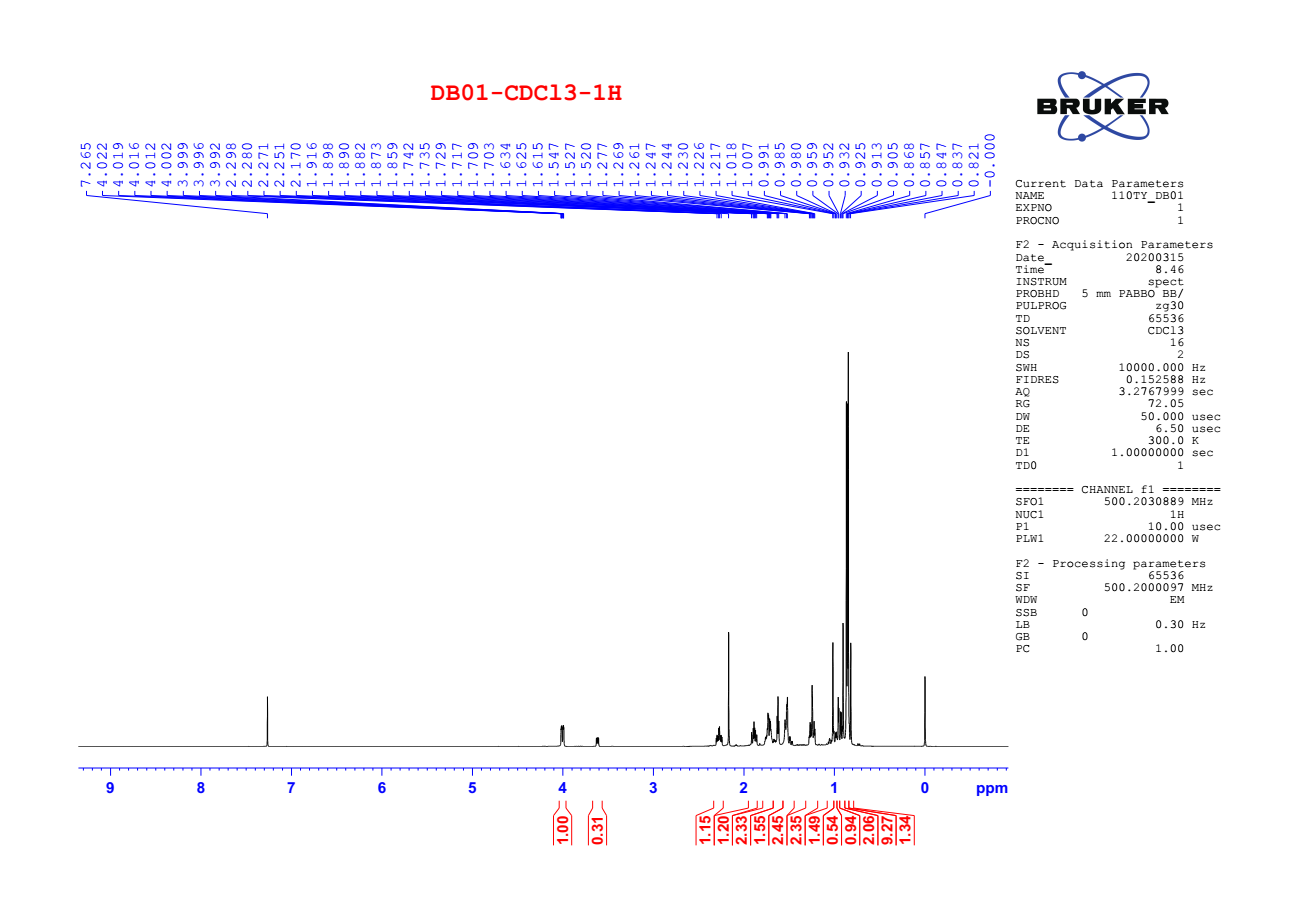
**

**Fig. S16** ^1^H-NMR spectrum (CDCl_3_, 500 MHz) of compound (**3**)

**
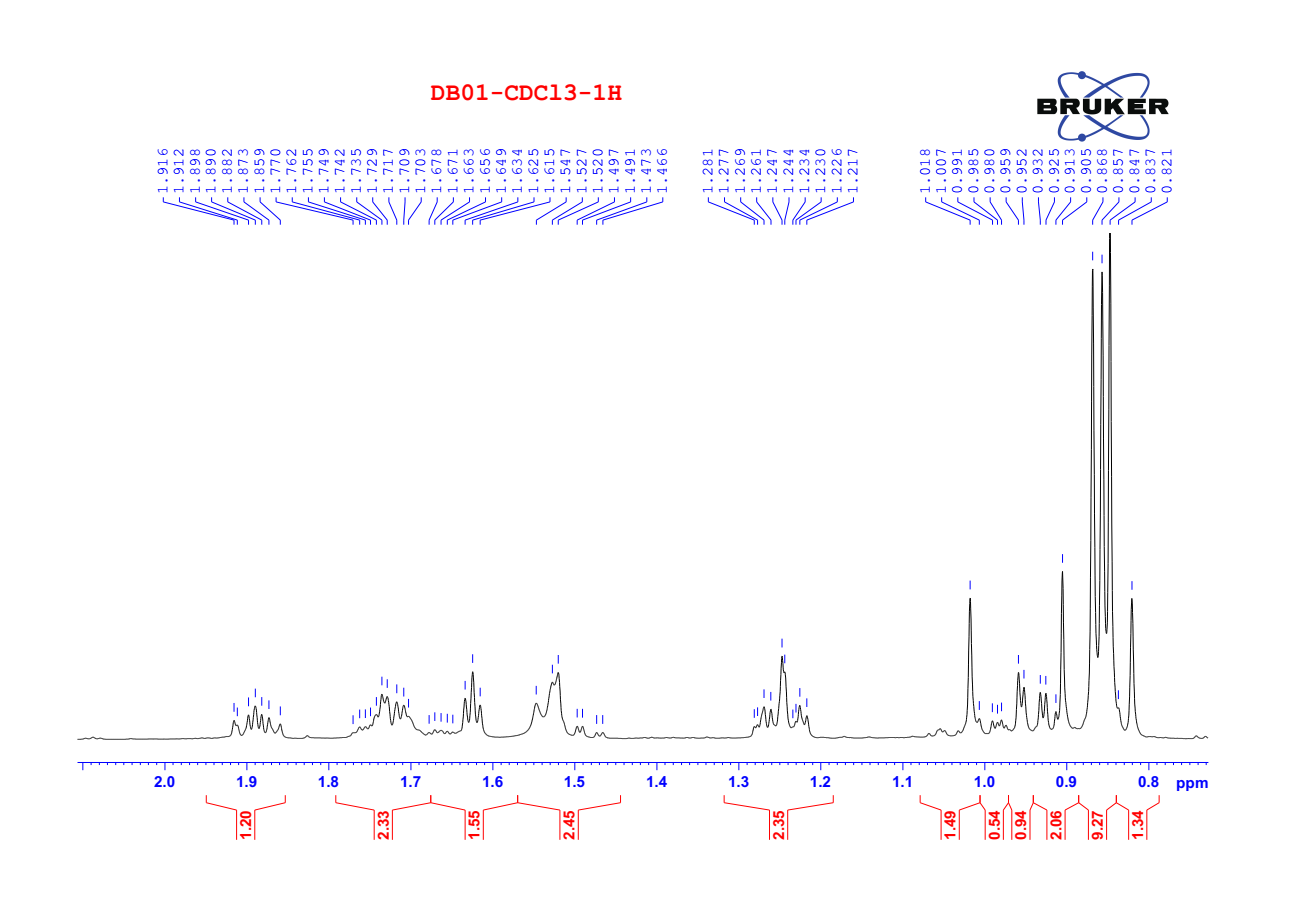
**

**Fig. S17** ^1^H-NMR spectrum (CDCl_3_, 500 MHz) of compound (**3**) (expansion 1)

**
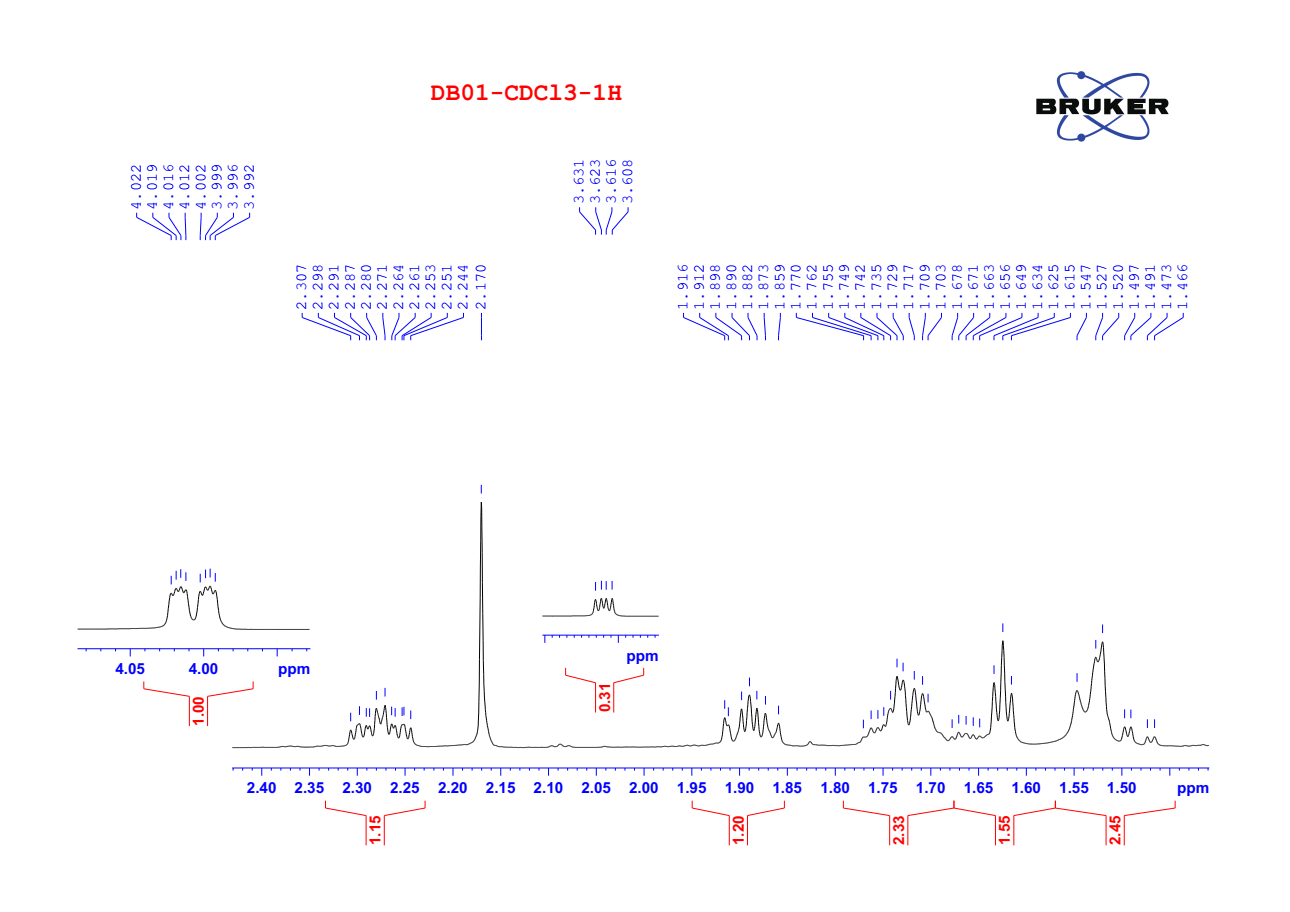
**

**Fig. S18** ^1^H-NMR spectrum (CDCl_3_, 500 MHz) of compound (**3**) (expansion 2)

**
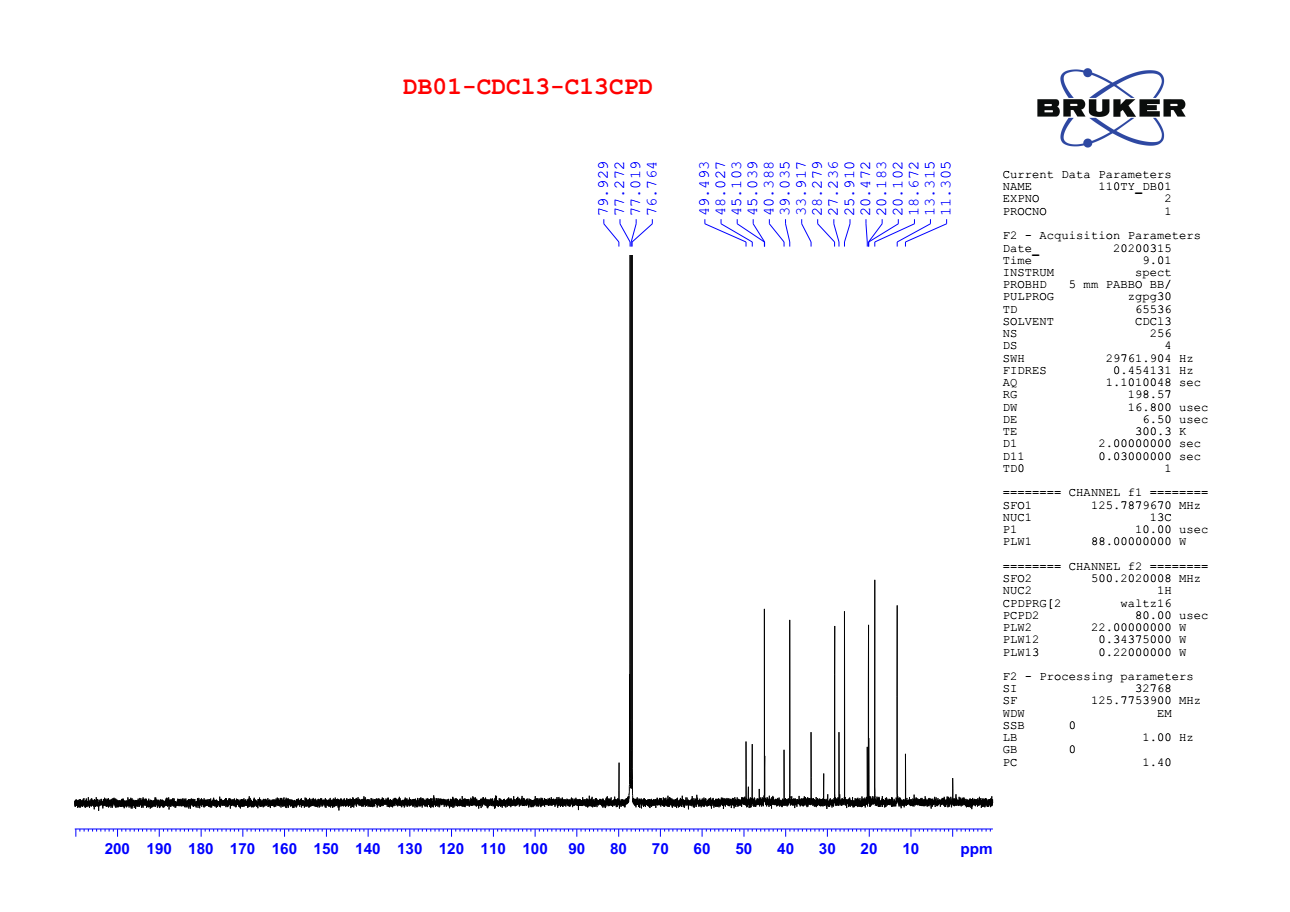
**

**Fig. S19** ^13^C**-**NMR spectrum (CDCl_3_, 125 MHz) compound (**3**)

**
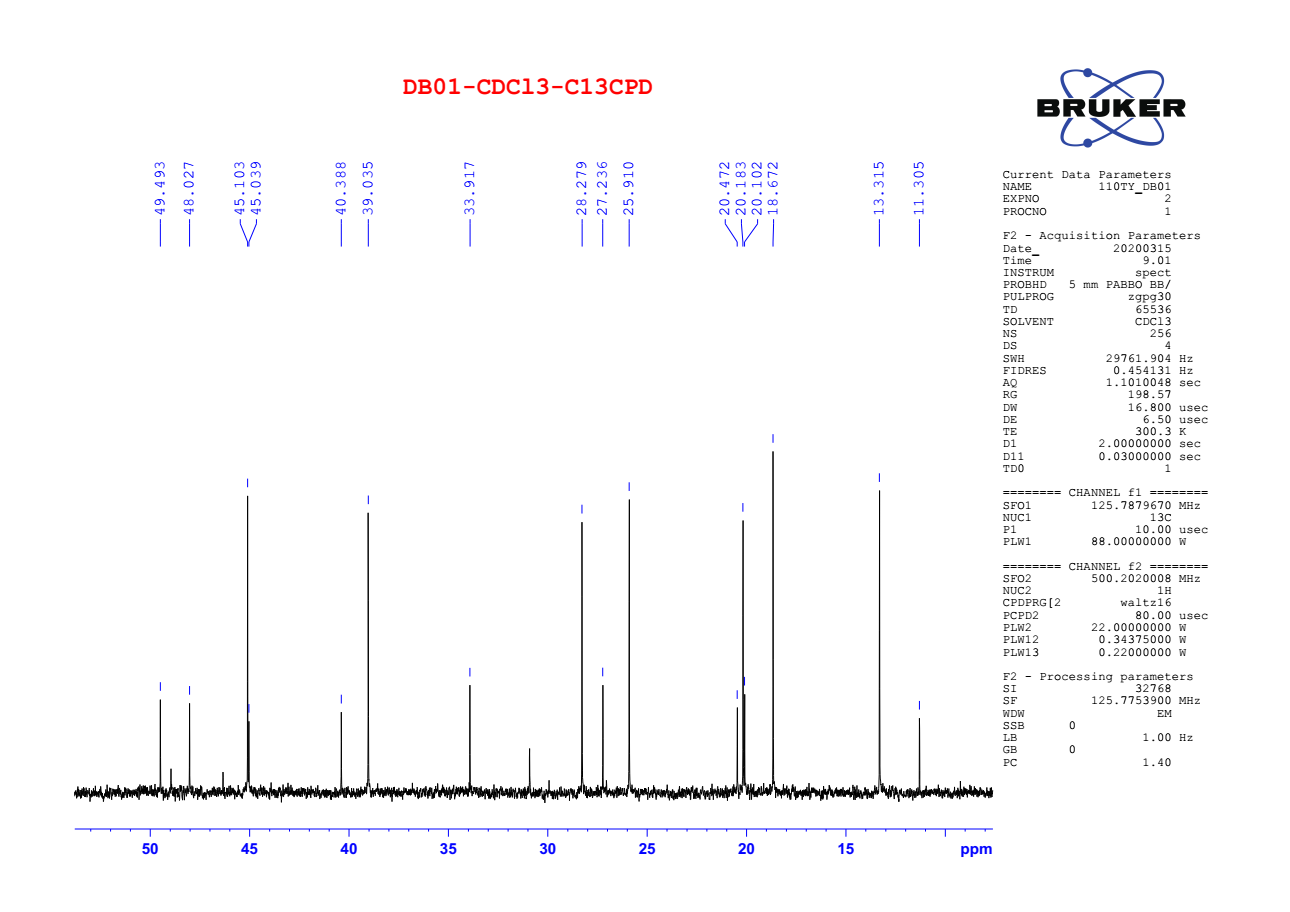
**

**Fig. S20** ^13^C**-**NMR spectrum (CDCl_3_, 125 MHz) compound (**3**) (expansion 1)

**
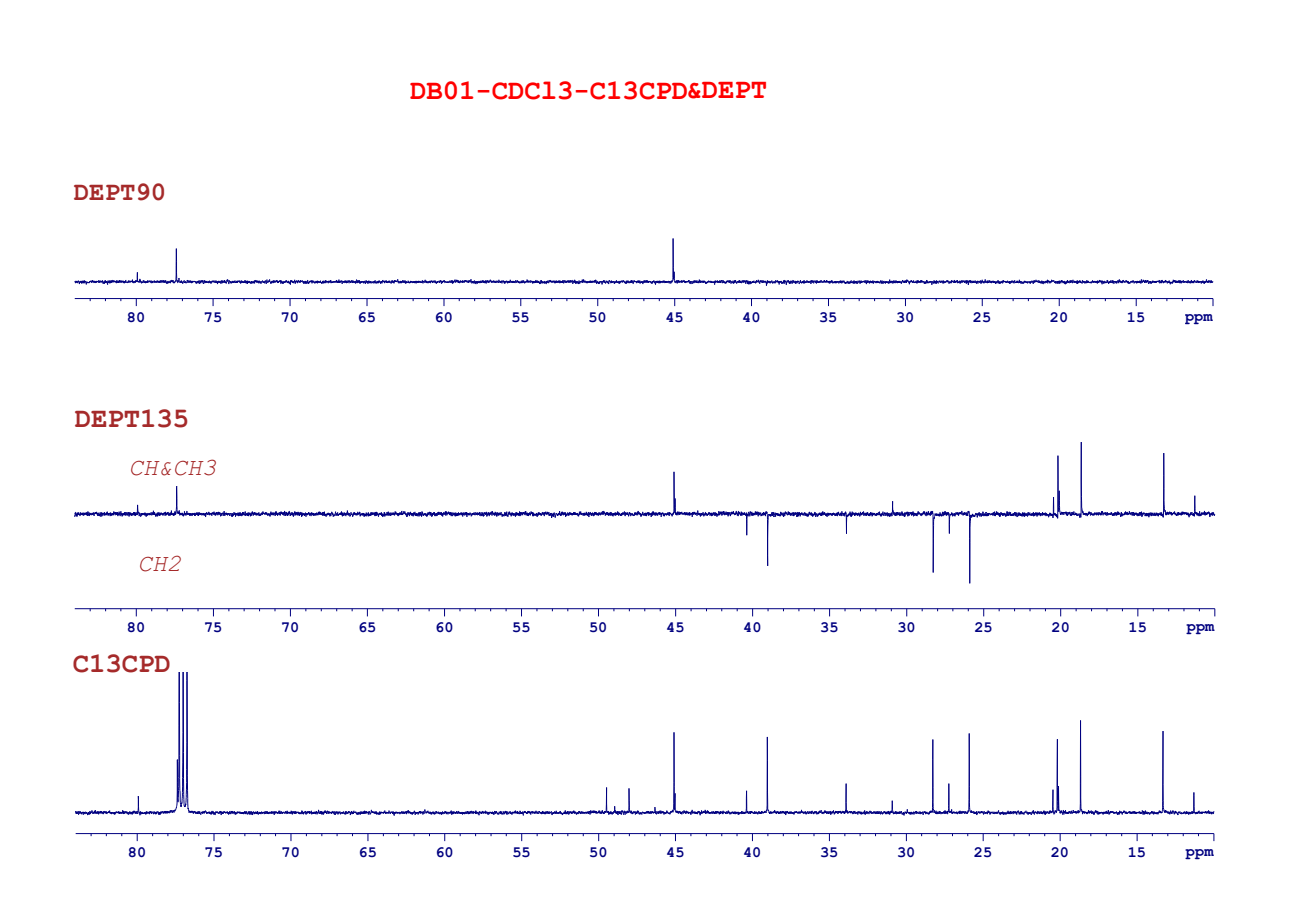
**

**Fig. S21** DEPT spectrum (CDCl_3_, 125 MHz) compound (**3**)

**
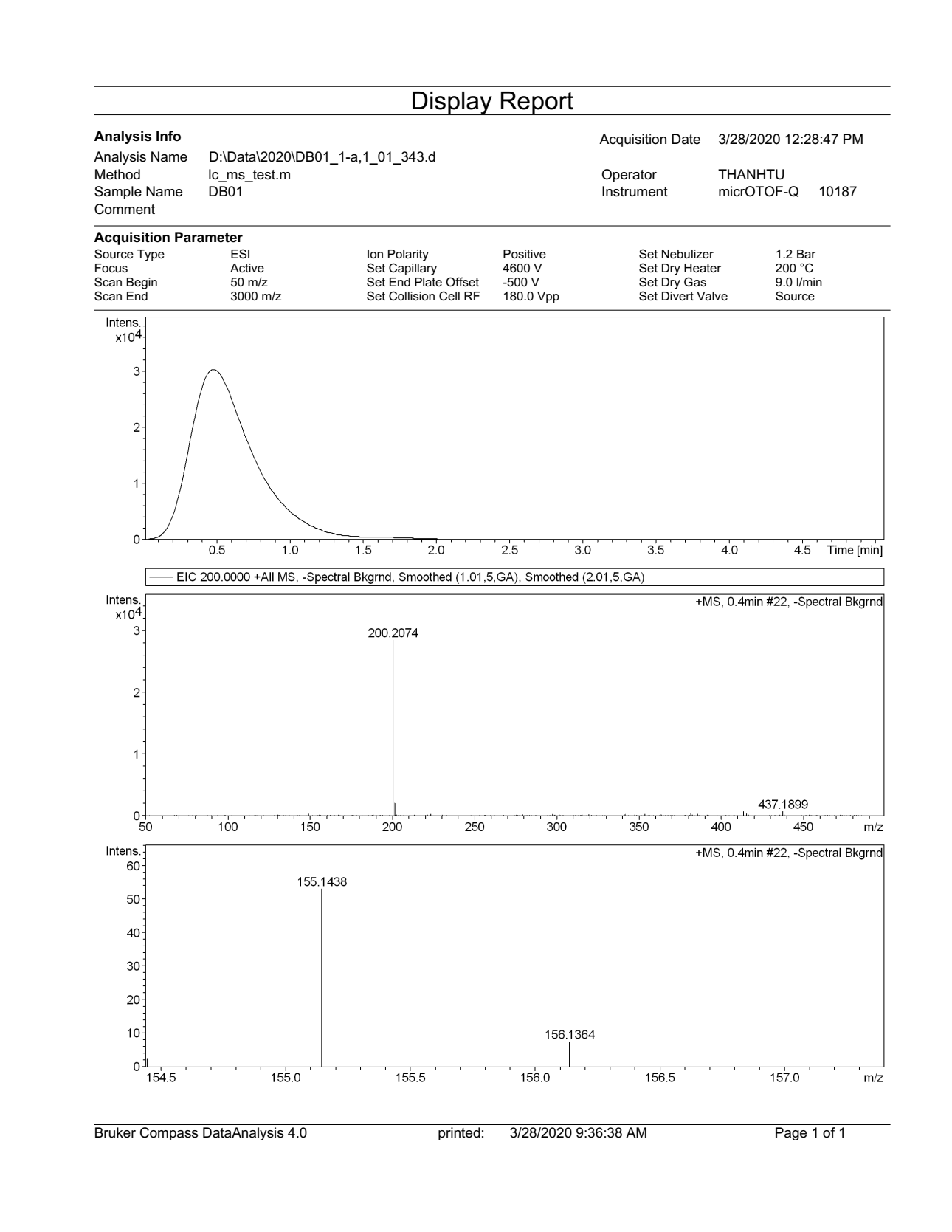
**

**Fig. S22** HRESIMS spectrum of compound (**3**)

| 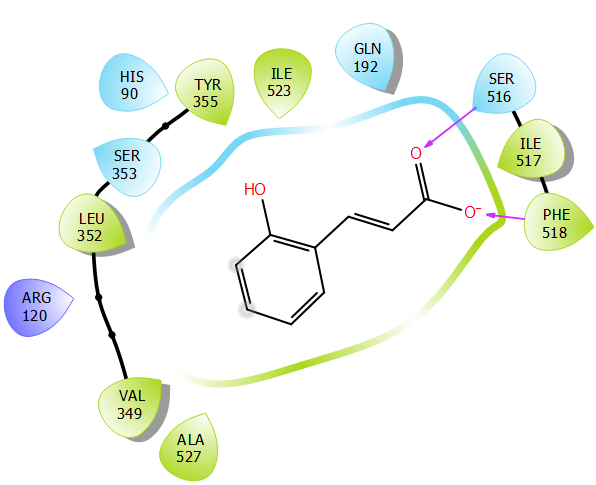 | 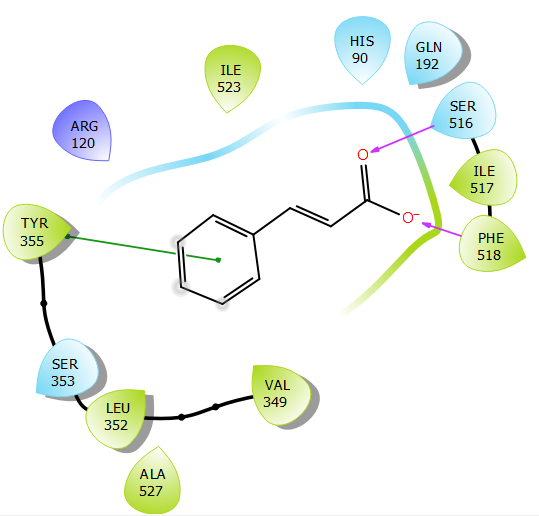 |
| --- | --- |
| 1 | 2 |
| 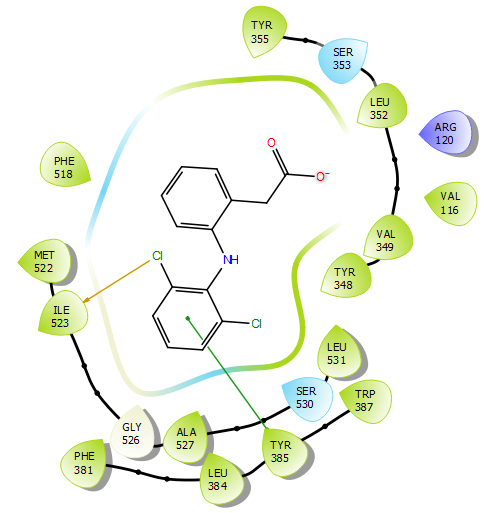 | |
| Diclofenac |  |

**Fig. S23** Binding poses of three molecule **1**, **2** and the diclofenac with COX-1 proteins. The additional informations in 2D diagrams present:


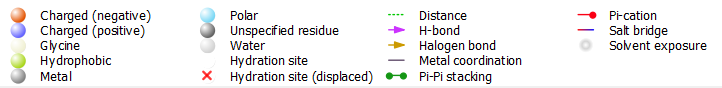

Supplement: Supplementary Materials — Figure S1: FT-IR spectrum (KBr) of compound (1). Figure S2: 1H-NMR spectrum (MeOD, 500 MHz) of compound (1). Figure S3: 1H-NMR spectrum (MeOD, 500 MHz) of compound (1) (expansion 1). Figure S4: 13C-NMR spectrum (MeOD, 125 MHz) compound (1). Figure S5: 13C-NMR spectrum (MeOD, 125 MHz) compound (1) (expansion 1). Figure S6: DEPT spectrum (MeOD, 125 MHz) compound (1) (expansion 1). Figure S7: HRESIMS spectrum of compound (1). Figure S8: FT-IR spectrum (KBr) of compound (2). Figure S9: 1H-NMR spectrum (MeOD, 500 MHz) of compound (2). Figure S10: 1H-NMR spectrum (MeOD, 500 MHz) of compound (2) (expansion 1). Figure S11: 13C-NMR spectrum (MeOD, 125 MHz) compound (2). Figure S12: 13C-NMR spectrum (MeOD, 125 MHz) compound (2) (expansion 1). Figure S13:. DEPT spectrum (MeOD, 125 MHz) compound (2). Figure S14: HRESIMS spectrum of compound (3). Figure S15: FT-IR spectrum (KBr) of compound (3). Figure S16: 1H-NMR spectrum (CDCl3, 500 MHz) of compound (3). Figure S17: 1H-NMR spectrum (CDCl3, 500 MHz) of compound (3) (expansion 1). Figure S18: 1H-NMR spectrum (CDCl3, 500 MHz) of compound (3) (expansion 2). Figure S19: 13C-NMR spectrum (CDCl3, 125 MHz) compound (3). Figure S20: 13C-NMR spectrum (CDCl3, 125 MHz) compound (3) (expansion 1). Figure S21: DEPT spectrum (CDCl3, 125 MHz) compound (3). Figure S22: HRESIMS spectrum of compound (3). Figure S23: Binding poses of three molecule 1, 2 and the diclofenac with COX-1 proteins. [file 6624347.f1.doc]
